# Targeting NCAM1/FGFR1 Signaling to Alleviate Sleep Disturbance-Induced Neuroinflammation in the Hypothalamus

**DOI:** 10.3390/cells15181716

**Published:** 2026-09-21

**Authors:** Yanxiang Qu, Bo Li, Shixuan Yan, Chuanjie Zhang, Guohua Ji, Kai Li, Yujie Zhao, Zuoyang Wang, Xiaopeng Li, Bo Song, Lina Qu

**Affiliations:** 1 Department of Pathology and Forensics, Dalian Medical University, Dalian 116044, China; qyx0902@163.com; 2 State Key Laboratory of Space Medicine, China Astronaut Research and Training Center, Beijing 100094, China; libook111@163.com (B.L.); 15052739230@163.com (S.Y.); zhangchuanjie96@163.com (C.Z.); jgh1682004@126.com (G.J.); albert_likai1993@163.com (K.L.); zhaoyujie9797@163.com (Y.Z.); 15846119800@163.com (Z.W.); lixiaopeng_acc@163.com (X.L.)

**Keywords:** sleep disturbance, neuroinflammation, hypothalamus, activation of microglia, inhibitory neurons, NCAM1/FGFR1 interaction

## Abstract

**Highlights:**

**What are the main findings?**

- Sleep disturbance (SD) induces neuroinflammation and neuronal injury in the hypothalamus, triggering microglial activation and facilitating a pro-inflammatory phenotypic transformation.
- SD selectively enhances pro-inflammatory responses and functional impairment in multiple hypothalamic inhibitory neuron subpopulations.

**What are the implications of the main findings?**

- NCAM1/FGFR1 signaling curbs microglial neuroinflammation triggered by SD, suggesting this signaling axis as an endogenous protective mechanism against SD-induced hypothalamic injury.
- Targeting the NCAM1/FGFR1 signaling pathway may represent a potential therapeutic strategy for alleviating neuroinflammation and neuronal dysfunction associated with sleep disorders.

**Abstract:**

Sleep disturbance is a prevalent public health concern associated with cognitive deficits and emotional dysregulation, yet the mechanisms underlying SD-induced hypothalamic dysfunction remain incompletely understood. Here, we found that two weeks of SD induced marked hypothalamic neuroinflammation, accompanied by increased expression of the pro-inflammatory cytokines *IL-1β*, *IL-6*, and *TNF-α*, as well as the chemokine *Ccl2*, together with reduced neuronal markers, including decreased Nissl-body and NeuN^+^ cells. Single-nucleus RNA sequencing (snRNA-seq) suggested that inhibitory neurons exhibited prominent transcriptional responses to SD, including enrichment of inflammatory pathways such as TNF and IL-17 signaling and transcriptional programs associated with neuronal dysfunction. Furthermore, SD was also associated with microglial activation and a phenotypic shift toward a disease-associated state, exhibiting enhanced antigen-presenting and pro-inflammatory capacity. Notably, intercellular communication analysis identified the neural cell adhesion molecule 1 (NCAM1)/fibroblast growth factor receptor 1 (FGFR1) signaling axis as a key mediator of crosstalk between microglia and other cell types. In BV2 cells, recombinant NCAM1 attenuated lipopolysaccharide-induced inflammatory responses in an *Fgfr1*-dependent manner. Consistently, PVN-targeted *Fgfr1* knockdown in vivo exacerbated SD-associated microglial activation, neuroinflammation, and neuronal injury. Collectively, our findings elucidate a critical protective role for the NCAM1/FGFR1 axis in mitigating SD-induced hypothalamic neuroinflammation and neuronal injury. This highlights this pathway as a candidate mechanism for further therapeutic investigation in sleep-related neurological dysfunction.

## 1. Introduction

Sleep is a fundamental biological process essential for brain homeostasis, cognitive function, and overall health [1,2,3]. However, in contemporary society, changes in lifestyle and the growing burden of sleep-related diseases have contributed to the increasing prevalence of sleep disorders, notably insomnia and obstructive sleep apnea [4,5], as classified by the International Classification of Sleep Disorders, Third Edition [6]. Accumulating evidence has indicated that SD is associated with a wide range of detrimental consequences, including adverse effects on cardiovascular health [2], altered immune function [7,8], and cognitive decline [9,10]. In particular, sleep loss has been linked to impaired clearance of proteins and metabolites from the central nervous system (CNS) [11], elevated production of pro-inflammatory cytokines such as interleukin-1 (IL-1) and tumor necrosis factor (TNF) [12], and increased oxidative stress [13]. These changes are also associated with microglial activation and neuroinflammation across several brain areas [14,15,16], processes that may contribute to neuronal injury and dysfunction [17,18]. Nonetheless, the mechanisms by which SD modulates neuroimmunity and neuronal integrity remain incompletely understood.

The hypothalamus, a critical brain region responsible for sleep–wake regulation, cardiovascular regulation, energy homeostasis, and neuroendocrine integration [19,20,21], is of particular interest in the context of SD. Growing evidence implicates neuroinflammation as a key mediator of SD-induced brain pathology [22,23]. Prolonged wakefulness can activate glial cells [24,25,26], particularly microglia, the resident immune cells of the CNS, leading to the release of inflammatory cytokines and chemokines. This neuroinflammatory milieu has been thought to contribute to synaptic remodeling [24], brain oxidative stress [25], abnormal protein deposition [26], decreased number of neurons [26], and altered neuronal excitability [27,28], which may collectively contribute to neuronal impairment. While prior studies have shown increased inflammatory markers in the brains of rodent models following SD, a comprehensive, cell-type-specific analysis of hypothalamic responses to SD is lacking. Identifying which neural cell types are primary drivers and targets of SD-induced inflammation is crucial for developing targeted interventions.

Neuronal injury in SD is closely linked to aberrant microglial activation and the resulting neuroinflammation [24,25,26]. Recent evidence has moved beyond the classical M1/M2 polarization framework, recognizing that microglia adopt a spectrum of activation states along a functional continuum [29,30]. According to single-cell transcriptome studies, microglial heterogeneity is shaped by both disease context and local microenvironmental cues, with distinct transcriptional programs associated with homeostatic surveillance, phagocytic activity, and pro-inflammatory responses [31,32]. In SD models, microglia have been shown to exhibit increased synaptic engulfment and elevated expression of inflammatory mediators, both of which directly impair neuronal function and survival [24,26,33]. Therefore, further exploration into the mechanism linking microglial activation, the production of inflammatory factors, and neuronal injury is warranted.

In the current study, hypothalamic impairment was found to be associatied with microglial neuroinflammation, which was governed by specific signaling pathways. In the hypothalamus, two-week SD caused robust neuroinflammation and progressively reduced neuronal markers. snRNA-seq analysis suggested that a specific neuronal cluster may be particularly vulnerable, exhibiting pronounced pro-inflammatory and neurodegenerative signatures. Furthermore, SD was associated with a phenotypic shift in microglia toward a disease-associated state with enhanced immune activation. Mechanistically, we identified NCAM1/FGFR1 as a candidate signaling pathway involved in the regulation of microglial inflammatory responses; we found altered downstream signaling associated with this pathway during SD. NCAM1 suppressed inflammatory activation in vitro in an *Fgfr1*-dependent manner, whereas PVN-targeted *Fgfr1* knockdown exacerbated SD-associated microglial activation, neuroinflammation, and neuronal injury in vivo. We therefore hypothesized that SD alters the protective signaling associated with the NCAM1/FGFR1 pathway, thereby contributing to microglial inflammatory activation and hypothalamic injury. These results point to a potential link between NCAM1/FGFR1 signaling and microglial homeostasis, which supports further investigation of this pathway as a candidate mechanism and potential therapeutic target in SD-associated hypothalamic impairment.

## 2. Materials and Methods

### 2.1. Sleep Disturbance Animal Model

The automated sleep interruption apparatus (SIA), as described in previous research, was utilized to induce SD [34]. A collection of 8-week-old male Sprague–Dawley rats purchased from SPF Biotechnology (Beijing, China) were housed under standard conditions (22 ± 2 °C, 50 ± 10% humidity, 12 h light/dark cycle) with food and water ad libitum. To minimize nonspecific stress associated with novelty and handling, rats assigned to the apparatus were acclimated to the SIA for 3 days before the formal SD procedure. SD was applied between ZT0 and ZT12 daily over a two-week period. The SIA parameters were set according to previous studies as follows: rotation speed at 60 s per revolution, and a rotation frequency of 1 min of rotation followed by a 2 min pause [35]. This relatively slow and intermittent rotation paradigm was designed to interrupt sustained sleep while avoiding continuous vigorous locomotor stimulation. In addition, no rotational stimulation was applied during ZT12–ZT24, providing the animals with a daily recovery period. After the two-week SD protocol, rats were deeply anesthetized by intraperitoneal injection of sodium pentobarbital (100–150 mg/kg) and subsequently euthanized by exposure to 70% CO_2_ in a thermostatically controlled chamber. Hypothalamic tissues were rapidly dissected and immediately transported to Beijing Berry Biotech (Beijing, China) for snRNA-seq.

The eight-week-old male C57BL/6J *mice* were obtained from SPF Biotechnology (Beijing, China) and were randomly divided into three groups: Ctrl (control), 1-week SD, and 2-week SD. All mice except those in the Ctrl group underwent the same SD protocol as described for rats. After the intervention, mice were euthanized via cervical dislocation without prior anesthesia. Hypothalamic tissues were rapidly dissected and snap-frozen in liquid nitrogen (n = 7 per group). For histopathological evaluation, whole brains were fixed in 4% paraformaldehyde for 24 h (n = 3 per group).

All animal procedures were performed in accordance with the institutional guidelines of the China Astronaut Research and Training Center and approved by its Institutional Animal Care and Use Committee (Approval No: ACC-IACUC-2024-012). All procedures were performed in accordance with the ARRIVE guidelines and regulations.

### 2.2. snRNA-seq of the Hypothalamus in Rats

Fresh hypothalamic tissues were obtained from the Ctrl and SD groups at 2 weeks post-treatment. Hypothalamic tissues were dissected from fresh brains based on anatomical landmarks [36,37]. The hypothalamic tissue block was dissected from the optic chiasm rostrally to the mammillary bodies caudally. The lateral boundaries were defined by the hypothalamic sulci, and the dorsal boundary was defined by the hypothalamic sulcus separating the hypothalamus from the thalamus. The dissected hypothalamic tissue was immediately flash-frozen in liquid nitrogen and stored at −80 °C until nuclei isolation for snRNA-seq. Following immediate collection, tissues were flash-frozen in liquid nitrogen to preserve RNA integrity. For sample preparation, three animals per condition were pooled to minimize sample variation [38]. All samples were subsequently transported on dry ice to Beijing Berry Biotech for snRNA-seq (10× Genomics Chromium platform). Single-nucleus suspensions were prepared using a microfluidics-based droplet method. Following nucleus isolation, cDNA synthesis, library construction, and quality assessment were performed according to the manufacturer’s protocol. Libraries were subsequently sequenced on an Illumina platform, and the resulting data were processed using standard snRNA-seq bioinformatic methods.

### 2.3. Pseudotime Trajectory

Cellular pseudotime inferred trajectories were reconstructed to infer state transitions using the Monocle 2 package (v.2.8.0). Cells were ordered along the trajectories based on a gene set identified by the differential gene test function with the following thresholds: mean expression ≥ 0.125, expressed in ≥10 cells, and a q-value < 0.01. The paths were visualized in two-dimensional t-SNE embeddings.

### 2.4. Gene Set Enrichment Analysis (GSEA)

To perform GSEA on our snRNA-seq data, we used the GSEA software v1.0 [39]. Gene sets were obtained from the Molecular Signatures Database (MSigDB), which includes hallmark gene sets, curated canonical pathways, and Gene Ontology gene sets. The input comprised a pre-ranked gene list ordered by log_2_ fold-change values derived from the differential expression analysis. Normalized enrichment scores (NES) were calculated for each gene set, with statistical significance evaluated through permutation testing. Gene sets with a false discovery rate (FDR) < 0.05 were considered significantly enriched.

### 2.5. Gene Set Score

We applied Seurat’s AddModuleScore function (default parameters) to quantify the expression level of a predefined gene set in single cells [40]. This method calculates a score for each cell as the difference between the average relative expression of the target gene set and that of a control gene set, which is randomly sampled from expression-matched control genes across binned expression intervals to correct for abundance-related background noise. This approach captures heterogeneity in cellular states, functions, or pathway activities.

### 2.6. DEGs

DEGs were identified using the FindMarkers function (test.use = “presto”) from the Seurat package [41]. DEGs were defined as those with a *p*-value < 0.01 and log_2_ fold-change > 0.58. Functional enrichment analyses, including Gene Ontology (GO) and the Kyoto Encyclopedia of Genes and Genomes encyclopedia of genes and genomes (KEGG) pathway annotations, were performed in R based on the hypergeometric distribution, with a significance cut-off of *p* < 0.05.

### 2.7. Lipopolysaccharide (LPS) and Recombinant NCAM1 Protein Treatment

To examine the effect of NCAM1 (R&D Systems, USA, Minneapolis, MN) on LPS-induced inflammation in BV2 cells, cells at approximately 80% confluency were assigned to two treatment protocols. For the pro-inflammatory microglial state, cells were stimulated with 1000 ng/mL LPS alone for 4 h [42]. To examine the effect of NCAM1, cells were exposed to 100 ng/mL recombinant NCAM1 protein for 4 h, followed by co-treatment with 1000 ng/mL LPS and 100 ng/mL NCAM1 for an additional 4 h.

### 2.8. Cell Culture and Transfection

The murine microglial cell line BV2 was obtained from Procell (Wuhan, China), Cat. No. CL-0493, and maintained in the specific medium, Cat. No. CM-0493, from the same company at 37 °C in a 5% CO_2_ incubator. BV2 cells were seeded in 6-well plates and grown to 70–80% confluence. Transient transfection with an shRNA plasmid targeting Fgfr1 (MailGene China, Beijing, China) was performed using Lipofectamine 3000 (Thermo Fisher Scientific, Carlsbad, CA, USA) according to the manufacturer’s protocol. Total RNA was extracted from transfected cells to evaluate how *Fgfr1* knockdown influences the anti-inflammatory effects mediated by NCAM1 in BV2 cells.

### 2.9. Hematoxylin-Eosin (H&E) Staining

Whole-brain tissue samples were processed for paraffin embedding, sectioned at 4–5 μm thickness, and subjected to H&E staining. Briefly, sections were baked at 60 °C for 2 h, deparaffinized in xylene, and rehydrated through a graded ethanol series (100% to 70%). Nuclei were stained with hematoxylin (Sigma-Aldrich, St. Louis, MO, USA), differentiated in 0.5% hydrochloric acid–ethanol, and rinsed in tap water. Cytoplasmic staining was performed with eosin (Sigma-Aldrich, St. Louis, MO, USA). The sections were then dehydrated through an ascending ethanol series, cleared in xylene, and mounted with neutral gum under a coverslip for microscopic examination. H&E staining images are representative of three independent biological replicates, and all images were acquired and quantified by an investigator blinded to group allocation.

### 2.10. Nissl Staining

Paraffin sections from each group were deparaffinized in xylene and rehydrated through a descending ethanol series (100%, 100%, 75%) followed by distilled water. The sections were then immersed in toluidine blue solution (Solarbio, Beijing, China) for 5 min, rinsed briefly with water, and differentiated in 1% acetic acid under microscopic observation until the desired contrast was achieved. The differentiation was stopped by rinsing in distilled water. After air-drying, Nissl-stained neurons were imaged under a light microscope. The integrated optical density of staining in each group was quantified using ImageJ software. Nissl staining images are representative of three independent biological replicates, and all images were acquired and quantified by an investigator blinded to group allocation.

### 2.11. Immunofluorescence (IF) Staining

Multiplex IF staining was performed using the Pano 4-plex IHC Kit (Panovue, Beijing, China), adhering to the procedural guidelines of the kit for sample processing and fluorescence staining methods. The primary antibodies included IBA1 (1:300, WAKO, Osaka, Japan) and NeuN (1:400, CST, Danvers, MA, USA). Images were acquired using a confocal microscope (Leica, Wetzlar, Germany), and fluorescence intensity was quantified with ImageJ software (1.54s). IF staining images are representative of three independent biological replicates, and all images were acquired and quantified by an investigator blinded to group allocation.

### 2.12. Analysis of Microglial Morphology

Microglial morphology was analyzed using ImageJ software. High-magnification images were converted to binary format using built-in plugins. Following binarization, outlines were generated, and the images were skeletonized to quantify morphological parameters, including soma area, the number of endpoints, and the maximum branch length of microglial processes [43]. IF staining images are representative of three independent biological replicates, and all images were acquired and quantified by an investigator blinded to group allocation.

### 2.13. Stereotaxic Injections of Adeno-Associated Virus (AAVs)

The 8-week-old male C57BL/6J *mice* received either AAV9-shFgfr1-EGFP 5.01 × 10^12^ or the shCtrl group (AAV9-EGFP), purchased from Brain Case (Shenzhen, China), into the PVN of the hypothalamus. The mice were maintained under isoflurane anesthesia in the stereotaxic apparatus. A glass capillary containing a viral vector was lowered into the brain (anterior–posterior (AP): −0.7 mm; medial–lateral (ML): 0.3 mm from the bregma; dorsal–ventral (DV): −4.5 mm from the dura; 200 nL injection [44]). Viral constructs were injected at a rate of 50 nL/min for 10 min, and the injector remained in place for an additional 5 min before removal. Three weeks after virus injection, the mice were subjected to 2 weeks of SD under the same conditions as previously described.

### 2.14. Western Blotting

Cells were homogenized in lysis buffer (Huaxingbio, Beijing, China) supplemented with a protease inhibitor cocktail on ice for 30 min. The lysates were centrifuged at 13,000× *g* for 20 min at 4 °C, and the supernatant was collected. Protein concentration was determined using a BCA assay kit (Thermo Fisher Scientific, USA). Equal amounts of protein were mixed with SDS loading buffer, denatured at 95 °C for 10 min, and separated on 10% SDS-polyacrylamide gels. Proteins were then transferred onto nitrocellulose membranes (Merck Millipore, Burlington, MA, USA). After blocking with 5% skim milk (BD Difco, Franklin Lakes, NJ, USA) in TBST for 1 h at room temperature, membranes were incubated overnight at 4 °C with primary antibodies: rabbit anti-FGFR1 (1:1000; Bioss, China, Beijing), rabbit anti-α-Tubulin (1:1000; Proteintech, Wuhan, China). Following three washes with 1×TBST, membranes were incubated with HRP-conjugated secondary antibodies for 1 h at room temperature. Protein bands were visualized using an enhanced chemiluminescence (ECL) detection kit (Merck Millipore, Burlington, MA, USA). Band intensities were quantified by densitometry analysis using ImageJ software.

### 2.15. RT-qPCR

Total RNA was extracted from tissues or cells using Trizol reagent (Invitrogen, Carlsbad, CA, USA) and quantified with a NanoDrop 2000 spectrophotometer (Thermo Fisher Scientific, Waltham, MA, USA). Reverse transcription was performed with the TransScript^®^ First-Strand cDNA Synthesis SuperMix (TRANS, Beijing, China), and mRNA expression was determined by RT-qPCR with PerfectStart^®^ Green qPCR SuperMix (TRANS, Beijing, China). GAPDH served as the endogenous control for normalization; primer sequences are provided in Table A1.

### 2.16. Statistical Analysis

All statistical analyses were performed using GraphPad Prism (version 10.6) or R (version 4.2.0). For in vitro experiments, three independent biological replicates were performed; for all in vivo experiments, seven biological replicates were included. Data are expressed as mean ± standard error of the mean (SEM). Comparisons between two groups were performed using an unpaired Student’s *t*-test or the Mann–Whitney U test, as appropriate. Differences among multiple groups were assessed by one-way analysis of variance. A *p*-value of less than 0.05 was considered statistically significant.

## 3. Results

### 3.1. SD Induces Neuroinflammation and Neuronal Injury in the Hypothalamus of Mice

**Figure 1:**
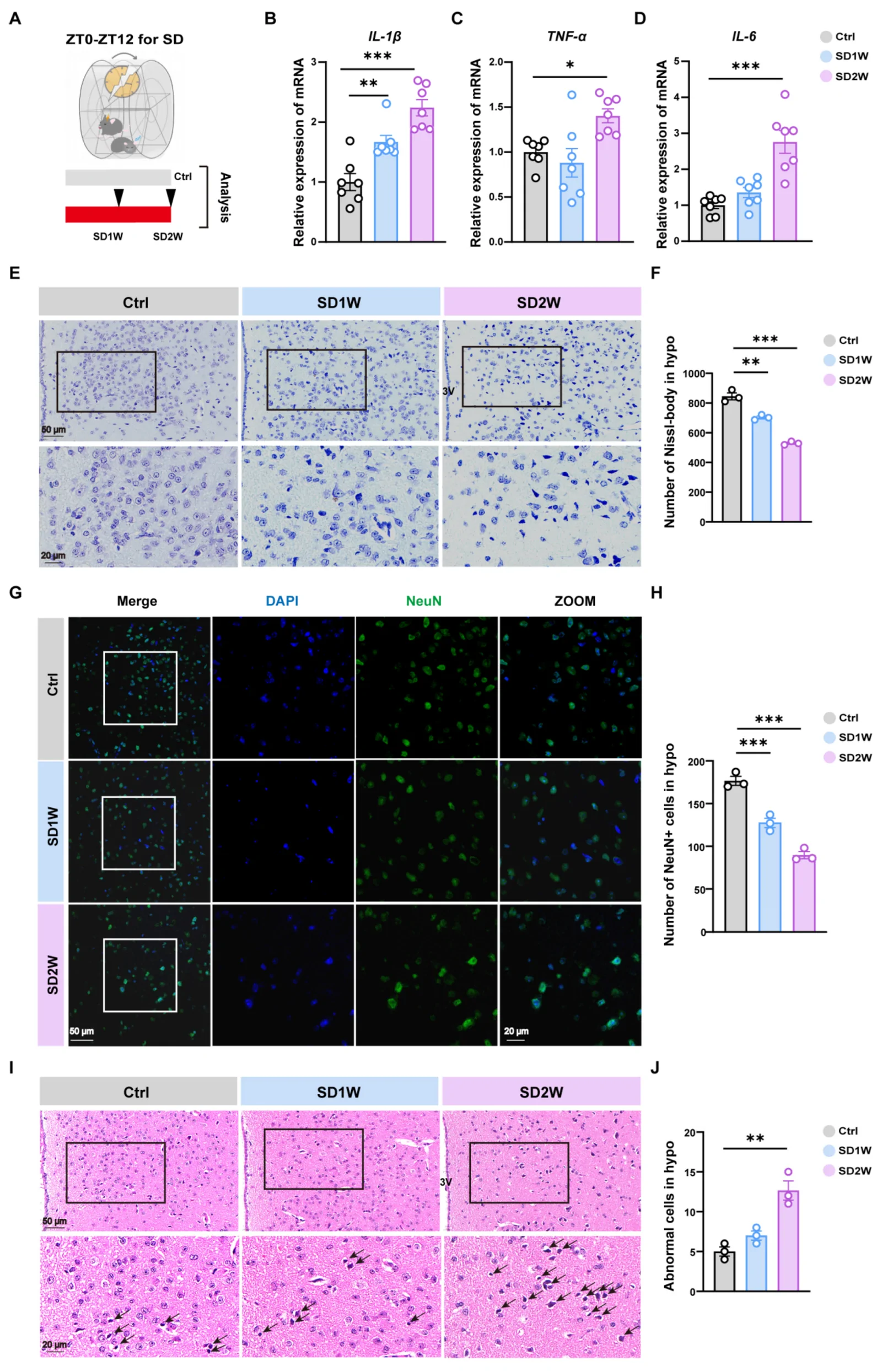
SD induces neuroinflammation and neuronal injury in the hypothalamus of mice. (**A**) Schematic representation of SD in mice. (**B**–**D**) mRNA expression levels of cytokine *IL-1β* (**B**), *TNF-α* (**C**), and *IL-6* (**D**) of the mouse hypothalamus in Ctrl, SD1W, and SD2W groups (n = 7/group). (**E**) Nissl staining of hypothalamic sections. Scale bars: upper panel, 50 μm, and lower panel, 20 μm. 3V, third ventricle. (**F**) Quantification of Nissl-body (n = 3/group). (**G**) Representative IF staining for NeuN+ cells. Scale bars: left panel, 50 μm, and right panel, 20 μm. (**H**) Quantitative analysis of NeuN+ cells (n = 3/group). (**I**) Representative H&E staining of hypothalamic sections. Arrows point to morphologically abnormal cells. (scale bars same as in (**E**)). (**J**) Semi-quantitative analysis of morphologically abnormal cells (n = 3/group). Data are presented as mean ± SEM. Significance is indicated as * *p* < 0.05, ** *p* < 0.01, and *** *p* < 0.001.

To investigate the effects of SD on neuroinflammation and neuronal reduced neuronal markers, adult male C57BL/6J mice were subjected to an orbital rotor-based SD paradigm daily during the ZT0-ZT12 period for 2 weeks (Figure 1A), with free access to water and food throughout.

We measured neuroinflammation marker levels in the hypothalamus across the experimental groups. Compared with the Ctrl mice, the SD2W mice exhibited markedly increased expression of multiple pro-inflammatory cytokines, *IL-1β*, *IL-6*, *TNF-α*, as well as the chemokine *Ccl2* in the hypothalamus (Figure 1B–D and Figure S1A). Only the inflammatory cytokine *IL-1β* was elevated in the SD1W group relative to the Ctrl group (Figure 1B). No significant changes were observed in the expression of the anti-inflammatory cytokines *IL-4*, *IL-10*, and *IL-13*, nor in the levels of *IL-17A* and *Cox2*, in either the SD1W or the SD2W groups (Figure S1B–F).

Histopathological analysis of the hypothalamus revealed progressive injury in this region following SD. To evaluate the impact of SD on neuronal integrity, we examined pathological changes in the hypothalamus using Nissl and IF staining. To evaluate neuronal integrity, Nissl staining was performed on brain sections from SD mice. The number of Nissl-body cells in the hypothalamus was slightly reduced in the SD1W group and markedly decreased in the SD2W group relative to the Ctrl group, indicating progressive neuronal impairment and reduced neuronal markers (Figure 1E,F). Consistently, IF staining for NeuN demonstrated a significant, time-dependent reduction in the number of NeuN+ neurons in the hypothalamus after SD (Figure 1G,H). Furthermore, H&E staining showed that, compared with the Ctrl mice, the number of abnormal hypothalamic cells was not significantly increased in the SD1W mice but was markedly elevated in the SD2W mice. These pathological alterations were characterized mainly by nuclear pyknosis and eosinophilic cytoplasmic degeneration (Figure 1I,J). Collectively, these findings indicated that SD induces hypothalamic neuroinflammation and progressive reduction in neuronal markers in mice.

### 3.2. Discovery-Oriented snRNA-seq in Rats Reveals That SD Induces Pro-Inflammatory Responses and Functional Impairment in Hypothalamic Inhibitory Neurons

**Figure 2:**
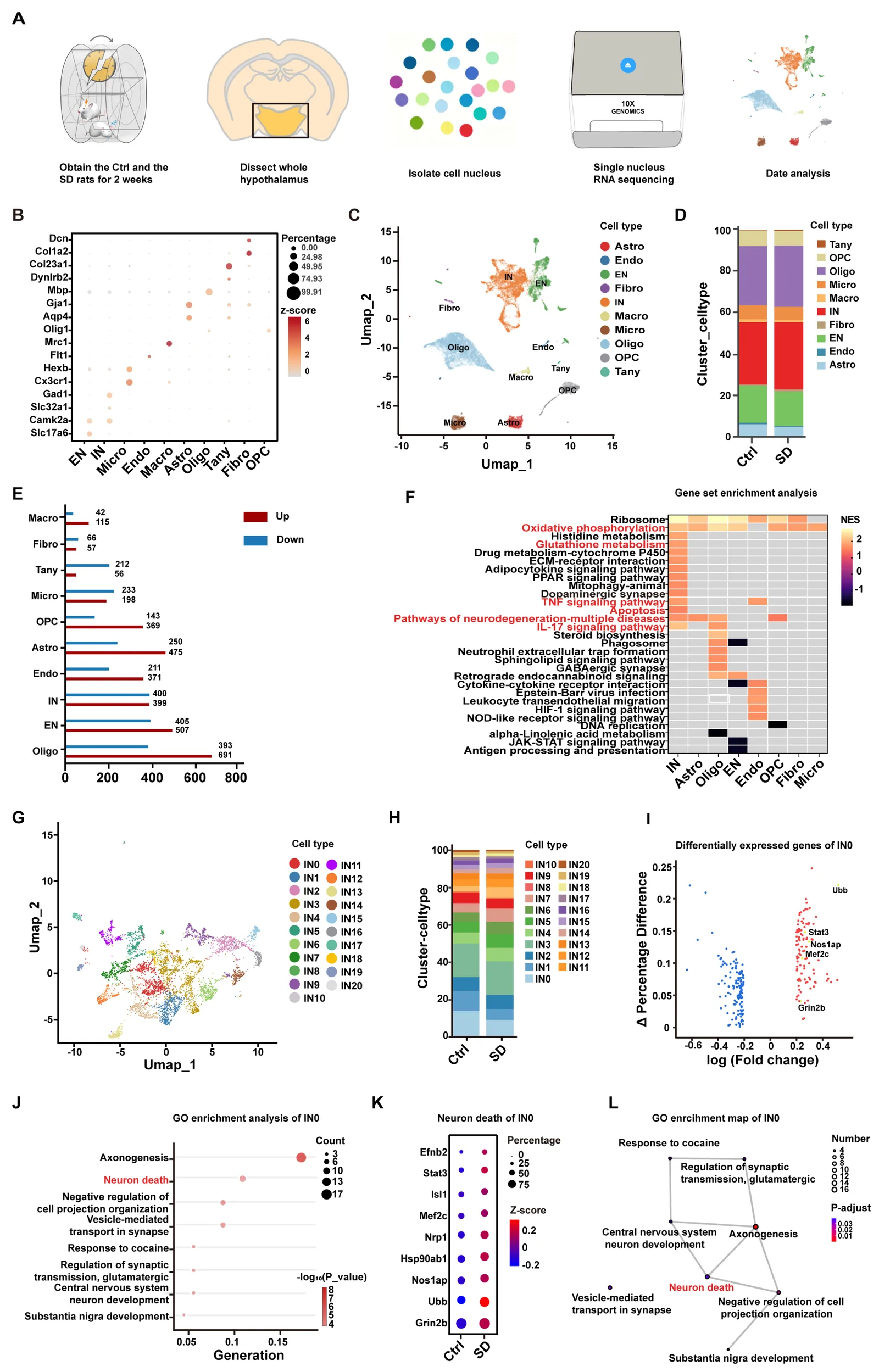
snRNA-seq uncovers inhibitory neuronal injury and dysfunction in the hypothalamus of rats. (**A**) Workflow of snRNA-seq on rat hypothalamus. (**B**) Bubble plot of marker gene expression across two neuronal and eight non-neuronal clusters. (**C**) Umap visualization of all 10 clusters (color-coded). (**D**) Cell proportion comparison of each cluster between Ctrl and SD groups. (**E**) Number of DEGs per cluster. (**F**) GSEA suggesting the enriched KEGG pathway in two neuronal and six non-neuronal clusters. (**G**) Umap of IN subclusters (color-coded). (**H**) The cell proportions of IN subclusters between Ctrl and SD groups. (**I**) DEGs of the IN0 cluster. (**J**) GO enrichment analysis of upregulated DEGs in the IN0 cluster for biological processes. (**K**) Bubble plot of neuronal death-related DEGs in IN0 between Ctrl and SD. (**L**) GO enrichment network map of upregulated DEGs in IN0.

As a discovery-oriented approach, snRNA-seq was performed on freshly isolated hypothalamic tissues from the Ctrl and the SD2W rats (Figure 2A) to identify candidate transcriptional alterations in specific hypothalamic cell types. A total of 20,497 nuclei were obtained from the two groups, with each condition represented by one pooled library derived from three rats. After semi-supervised clustering and manual quality control, 18,330 high-quality nuclei were retained and classified into 10 transcriptionally distinct clusters. Based on canonical marker expression, these clusters were annotated as two neuronal and eight non-neuronal clusters, including excitatory neurons (EN: *Slc17a6*, *Rorb*), inhibitory neurons (IN: *Slc32a1*, *Gad1*), astrocytes (Astro: *Gfap*, *Aqp4*), and microglia (Micro: *Cx3cr1*, *Hexb*, *P2ry12*) [45,46], etc. (Figure 2B). Umap analysis revealed an altered cellular landscape in the hypothalamus following SD (Figure 2C). Cell proportion analysis revealed a relative increase in the proportion of IN and a decrease in EN in the SD2W group relative to the Ctrl group (Figure 2D). However, because relative proportions in snRNA-seq data can be affected by technical biases (e.g., differential nuclear recovery or capture efficiency), these shifts should not be interpreted as absolute neuronal injury or gain. Nonetheless, they suggest a possible excitatory–inhibitory imbalance that warrants histological validation. DEG analysis identified 799 DEGs in IN (399 upregulated, 400 downregulated) and 912 DEGs in EN (507 upregulated, 405 downregulated), highlighting substantial transcriptomic remodeling within these clusters (Figure 2E).

To investigate cell-type-specific transcriptional perturbations induced by SD, GSEA was performed on each hypothalamic cell cluster. Among all cell clusters, IN exhibited the strongest transcriptional response. In this cluster, SD significantly enriched multiple pro-inflammatory pathways, including TNF signaling, IL-17 signaling, apoptosis, and neurodegeneration-related pathways. Pathways involved in energy metabolism (ribosome, oxidative phosphorylation, and mitophagy), oxidative stress (glutathione metabolism), and anti-inflammatory lipid metabolism (PPAR signaling and adipocytokine signaling) were also upregulated (Figure 2F). These findings indicated that IN responds to SD with a predominantly pro-inflammatory response dominated by TNF and IL-17 signaling, despite concurrent activation of the compensatory PPAR pathway. Compared with IN, oligodendrocyte (Oligo) and endothelial cells (Endo) showed more restricted but still notable responses. In Oligo, SD enriched pathways related to IL-17 signaling, neurodegeneration-multiple diseases, phagosome, neutrophil extracellular trap formation, steroid biosynthesis, sphingolipid signaling, GABAergic synapse, and retrograde endocannabinoid signaling (Figure 2F), suggesting concurrent inflammatory activation and lipid metabolic disturbance. In Endo, SD upregulated pathways involved in TNF, cytokine-cytokine receptor interaction, NOD-like receptor signaling, leukocyte transendothelial migration, and HIF-1 signaling (Figure 2F), consistent with endothelial activation and vascular inflammatory stress. By contrast, other cell types, including EN, Micro, Astro, oligodendrocyte precursor cells (OPC), and fibroblasts (Fibro), showed no clear enrichment of inflammatory pathways. Taken together, these findings suggested that SD elicits a cell-type-specific transcriptional response in the hypothalamus, with IN representing the most responsive and inflammation-associated cluster. Oligo and Endo cells also contribute to SD-associated neuroinflammatory and vascular alterations, respectively, whereas other cell clusters show relatively limited inflammatory activation.

Given the marked shift in neuronal composition in the hypothalamus after SD, we next characterized neuronal clusters. Using the identified marker genes for EN and IN, we generated a gene expression heatmap and further distinguished IN and EN within the neuronal clusters by Umap analysis (Figure S2A,B). To define the molecular features of SD in these neuronal clusters, we performed DEG analysis separately in EN and IN.

In EN, SD upregulated several stress-related genes (*Actb*, *Fth1*, *UBB*, *Hbb-b1*) and downregulated genes associated with genomic stability and transport (*Nek5*, *Slc5a4*, *Trrap*, *Rsrp1*, *Sdccag8*) (Figure S2C). GO analysis suggested activation of compensatory synaptic programs and impaired post-transcriptional regulation (Figure S2D). A similar but more pronounced transcriptional pattern was observed in IN. Stress-responsive genes, including *Cst3*, *Ubb*, *and Hbb-b1*, were increased, whereas genes involved in RNA splicing and transport, such as *Slc5a4*, *Pcbp2*, and *Sdccag8*, were reduced (Figure S2E,F). KEGG analysis revealed enrichment of neurodegeneration-related pathways, including the Alzheimer’s disease pathway, in both EN and IN, accompanied by suppression of ribosome-associated pathways (Figure S2G,H). These findings indicated that SD broadly impairs protein synthesis and promotes neurodegenerative transcriptional programs, with IN showing a particularly robust response.

To determine which inhibitory neuronal subclusters were most affected by SD, IN were further reclustered into 21 subclusters based on subcluster-specific marker genes (Figure 2G and Figure S3A). Cell proportion analysis showed that IN0 and IN1 were the most prominently reduced subclusters in the SD group compared with the Ctrl group (Figure 2H). As noted above, these proportional changes reflect relative abundance in the recovered nuclei and should be interpreted cautiously, rather than as definitive evidence of absolute subcluster loss. DEGs of IN0 showed upregulation of multiple genes associated with neuronal injury, including *Ubb*, *Stat3*, *Nos1ap*, *Mef2c*, and *Grin2c* (Figure 2I). The functions of the genes identified by snRNA-seq are described in Table A2. GO analysis further demonstrated significant enrichment of axonogenesis, neuron death, and negative regulation of cell projection organization in this subcluster (Figure 2J), and a bubble plot of neuron death-related genes further supported this pattern (Figure 2K). These results suggested that SD may activate a neurodegenerative program in IN0. Additional GO analysis identified several functionally related pathways associated with neuron death, including axonogenesis, CNS neuron development, and negative regulation of cell projection organization (Figure 2L and Figure S3B). Among the key genes, *Ubb* was enriched in CNS development and neuron death; *Nrp1* was linked to CNS development, neuron death, axonogenesis, and negative regulation of cell projection organization; and *Efnb2* was involved in vesicle-mediated transport in synapses, neuron death, negative regulation of cell projection organization, and axonogenesis. In addition, *Nrp1*, *Mef2c*, *Isl1*, and *Hsp90ab1* were repeatedly enriched across both neuron death- and axonogenesis-related pathways (Figure S3B). These findings suggested that multiple coordinated pathways, particularly those related to axonal remodeling and synaptic regulation, converged on the Ctrl group of neuron death in IN0.

IN1 also displayed marked transcriptional dysregulation after SD. DEGs showed that downregulated genes, including *Srsd7*, *Pnisr*, and *Snrnp7*, were mainly enriched in nuclear speck-associated pathways (Figure S3C). GO analysis further showed that pathways related to the nuclear speck and the SAGA complex were considerably suppressed in IN1 (Figure S3D). These findings suggested that SD disrupts alternative splicing homeostasis in IN1, which may have contributed to neuronal dysfunction by impairing the processing of genes involved in synaptic maintenance and cell survival. Notably, GSEA further demonstrated significant upregulation of the IL-17 signaling pathway in IN1 after SD compared with the Ctrl group (Figure S3E). Given the established pro-inflammatory role of IL-17 in the CNS, this result indicated that SD elicits a pronounced inflammatory response in this inhibitory neuronal subcluster.

Together, these findings supported the conclusion that IN represents a major target of SD-induced hypothalamic injury and undergoes both inflammatory activation and functional impairment.

### 3.3. SD Induces Hypothalamic Microglial Activation and Promotes a Pro-Inflammatory Phenotypic Shift

**Figure 3:**
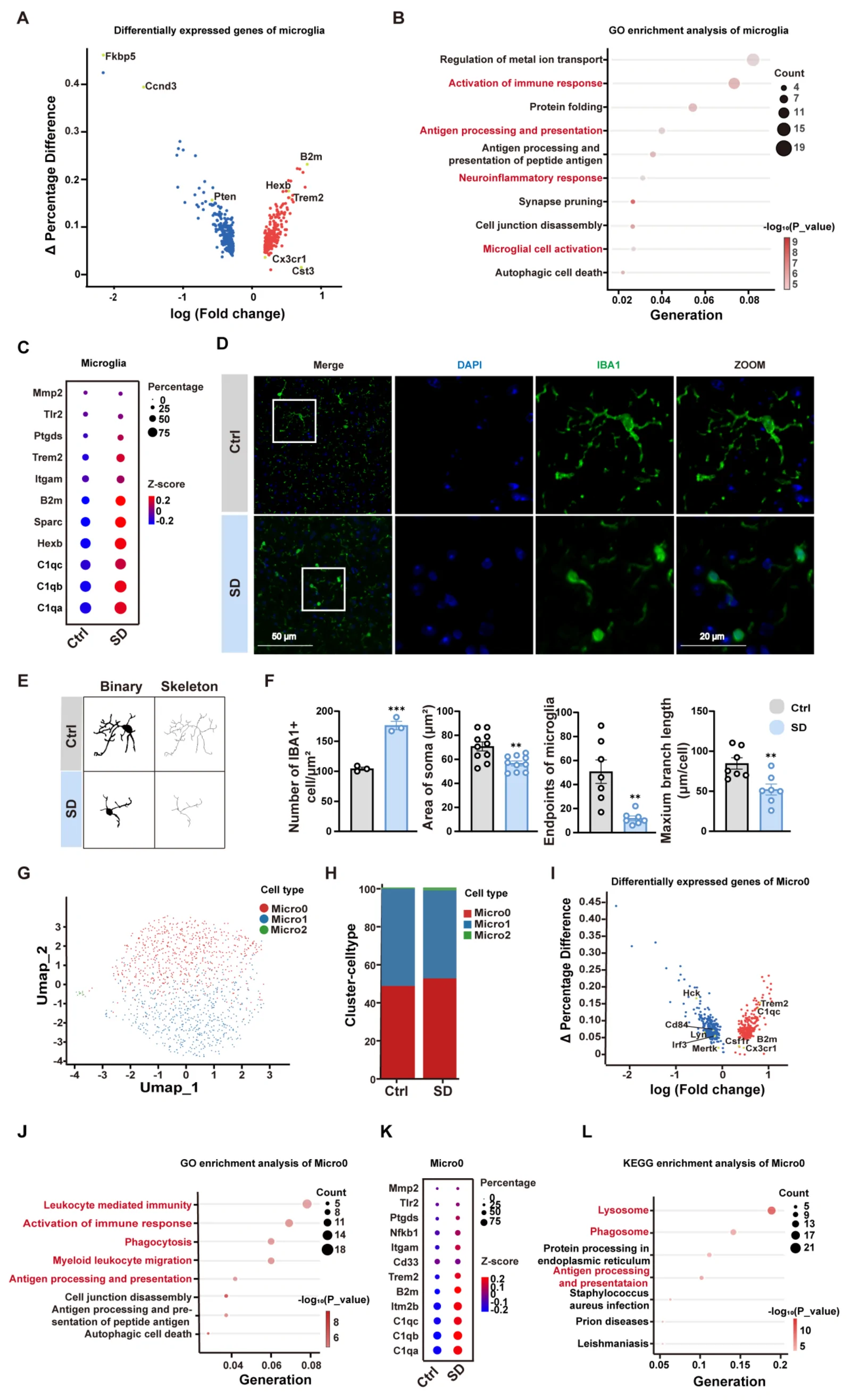
SD induces hypothalamic microglial activation and promotes a pro-inflammatory phenotypic shift. (**A**) DEGs of the microglia cluster. (**B**) GO enrichment of upregulated DEGs in microglia for BP. (**C**) Bubble plot of inflammation-related DEGs in microglia between Ctrl and SD groups. (**D**) Representative IF staining for IBA1+ cells (Scale bars: left panel, 50 μm, and right panel, 20 μm). (**E**) The binary and skeleton of microglia. (**F**) Diagram of quantification of microglia (n = 3/group). (**G**) Umap visualization of microglia subclusters (color-coded). (**H**) The cell proportions of microglia subclusters between Ctrl and SD groups. (**I**) DEGs of the Micro0 cluster. (**J**) GO enrichment analysis of upregulated DEGs in the Micro0 cluster for BP. (**K**) Bubble plot of inflammation-related DEGs in the Micro0 cluster between Ctrl and SD groups. (**L**) KEGG enrichment analysis of upregulated DEGs in the Micro0 cluster. Data are presented as mean ± SEM. Significance is indicated as ** *p* < 0.01, and *** *p* < 0.001.

To determine whether SD alters the state of hypothalamic microglia, we first analyzed microglial transcriptomic changes in the rat snRNA-seq dataset. DEGs revealed a distinct shift in the transcriptional profile of hypothalamic microglia in the SD group, with increased expression of *Cst3*, *Hexb*, and *Cx3cr1*, together with upregulation of disease-associated microglia (DAM)-related genes, including *Trem2*, *B2m*, and *Itgam*. In contrast, proliferation-related genes, including *Fkbp5* and *Ccnd3*, as well as *Pten*, were downregulated (Figure 3A). The upregulated genes in the Micro1 cluster were similar to those in the microglia cluster (Figure S4A). These results point to a significant reprogramming of hypothalamic microglia’s transcriptional status by SD.

GO enrichment analysis showed that the upregulated genes of microglia were mainly enriched in biological processes associated with activation of the immune response, neuroinflammatory response, antigen processing and presentation, and microglial cell activation after SD (Figure 3B). Consistently, the bubble plot analysis revealed that *C1qa*, *C1qb*, *C1qc*, and *Trem2* were predominantly enriched in immune response activation, whereas *B2m*, *Psap*, and *Ctss* were mainly associated with antigen processing and presentation. In addition, *C1qa*, *Trem2*, *Cx3cr1*, and *Itgam* were enriched in pathways related to microglial activation and neuroinflammation, while *Sparc*, *Ptgds*, *Mmp2,* and *Tlr2* were associated with neuroinflammatory responses (Figure 3C). Together, these data suggested that SD may promote microglial activation and a pro-inflammatory transcriptional program.

We next assessed whether SD-induced transcriptional alterations were accompanied by morphological changes in hypothalamic microglia. IF analysis showed that microglia in the SD group exhibited increased cell density and reduced branching complexity compared with the Ctrl group, as reflected by fewer branches and a shorter maximum branch length (Figure 3D–F). These morphological features are consistent with a shift toward an activated microglial phenotype.

To further advance the characterization of microglial heterogeneity after SD, we performed subcluster analysis of hypothalamic microglia. Umap visualization and the gene expression heatmap revealed distinct microglial subclusters (Figure 3G and Figure S4B), and cell proportion analysis revealed an increase in the Micro0 cluster within the SD group relative to the Ctrl group (Figure 3H). DEGs of the Micro0 cluster exhibited elevated expression levels of *C1qc*, *Trem2*, *B2m*, *Csf1r*, and *Cx3cr1*, together with reduced expression of *Hck*, *Cd84*, *Lyn*, *Irf3*, and *Mertk* (Figure 3I). This expression pattern indicated that the Micro0 cluster represents a microglial subset enriched after SD with DAM-like transcriptional features.

Functional annotation further showed that the genes upregulated in the Micro0 cluster were markedly enriched in leukocyte-mediated immunity, activation of immune response, phagocytosis, myeloid leukocyte migration, and antigen processing and presentation (Figure 3J). GO enrichment analysis indicated that the transcriptome profiles of the microglia cluster and the Micro1 cluster predominantly mirrored those of the Micro0 cluster (Figures S4C and S5A). The bubble plot analysis of the Micro0 cluster resembled that of microglia (Figure 3K). KEGG analysis showed significant enrichment of lysosome, phagosome, and antigen processing and presentation pathways in the Micro0 cluster (Figure 3L), and the KEGG enrichment results for the Micro1 and the microglia clusters (Figures S4D and S5B) largely mirrored those of the Micro0 cluster. GO enrichment analysis further revealed that SD induced downregulation of pathways associated with immune development (regulation of hemopoiesis and regulation of myeloid cell differentiation) and protein catabolism (regulation of proteolysis involved in cellular protein catabolic process, and regulation of ubiquitin-dependent protein catabolic process) in hypothalamic microglia (Figure S5C). The downregulation of proteostatic machinery suggested compromised protein quality control, potentially endangering microglial immune surveillance. Additionally, the reduced expression of erythrocyte-related pathways, while not directly linked to erythropoiesis in microglia, may suggest disrupted microglia-vascular interactions that contribute to hypothalamic dysfunction. These data suggest that SD may enrich a microglial subcluster with heightened immune activation and DAM-like pro-inflammatory features.

### 3.4. Pseudotime Trajectory Reveals Transcriptional Profiles Consistent with a DAM1-like State

**Figure 4:**
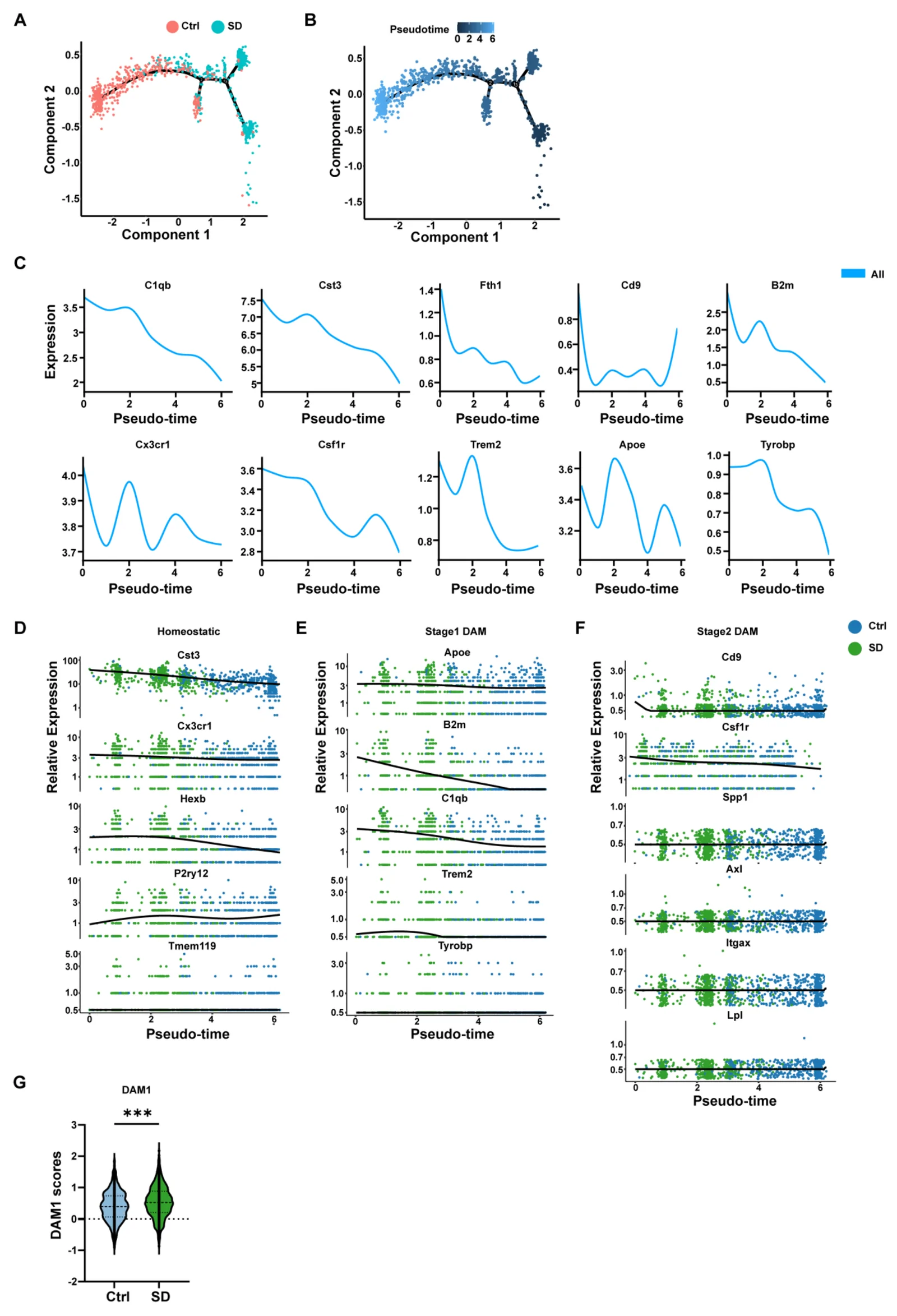
Pseudotime trajectory of microglia in the hypothalamus of rats. (**A**) Pseudotime trajectory of microglia between Ctrl and SD groups. (**B**) Pseudotime plot showing the inferred transcriptional ordering of all microglial cells between Ctrl and SD groups. (**C**) Expression dynamics of homeostatic microglia, DAM1, and DAM2 marker genes along pseudotime. (**D**–**F**) The scatter plots show the dynamic expression of genes related to homeostatic microglia (**D**), DAM1 (**E**), and DAM2 (**F**) along pseudotime. (**G**) DAM1 gene set scores between Ctrl and SD groups. Data are presented as mean ± SEM. Significance is indicated as *** *p* < 0.001.

To elucidate the dynamic trajectory of microglial state transitions under SD, microglia were classified into three distinct subclusters based on canonical marker genes: homeostatic microglia, identified by the expression of *Cx3cr1*, *P2ry12*, *Tmem119*, *Hexb*, and *Cst3*; DAM phase 1 (DAM1), characterized by *Cx3cr1*, *P2ry12*, *Tmem119*, *Tyrobp*, *Apoe*, *B2m*, and *Trem2*; and DAM phase 2 (DAM2), distinguished by *Lpl*, *Axl*, *Cd9*, *Spp1*, *Cst7*, and *Itgax*. Pseudotime analysis revealed distinct inferred transcriptional trajectories between the SD and the Ctrl groups (Figure 4A), accompanied by changes in the inferred ordering of cellular states (Figure 4B). Trajectory reconstruction using homeostatic and DAM marker genes suggested that the three functional states are computationally ordered along defined molecular pathways (Figure 4C), consistent with an inferred progression toward a DAM-like state. The scatter plot revealed that, in comparison to the Ctrl group, the expression levels of B2m and Trem2 were elevated in the SD group at DAM1 (Figure 4D–F). The expression of DAM2 microglial marker genes exhibited minimal changes along the pseudo-time, suggesting that DAM2 might not play a substantial role in hypothalamic microglial abnormalities after SD (Figure 4F). Notably, DAM1 scores were significantly higher in the SD group relative to the Ctrl group (Figure 4G). These findings suggested that SD is associated with transcriptional profiles that are consistent with an inferred trajectory toward a DAM1-like state.

### 3.5. The NCAM1/FGFR1 Pathway Negatively Regulates Microglial Inflammation

**Figure 5:**
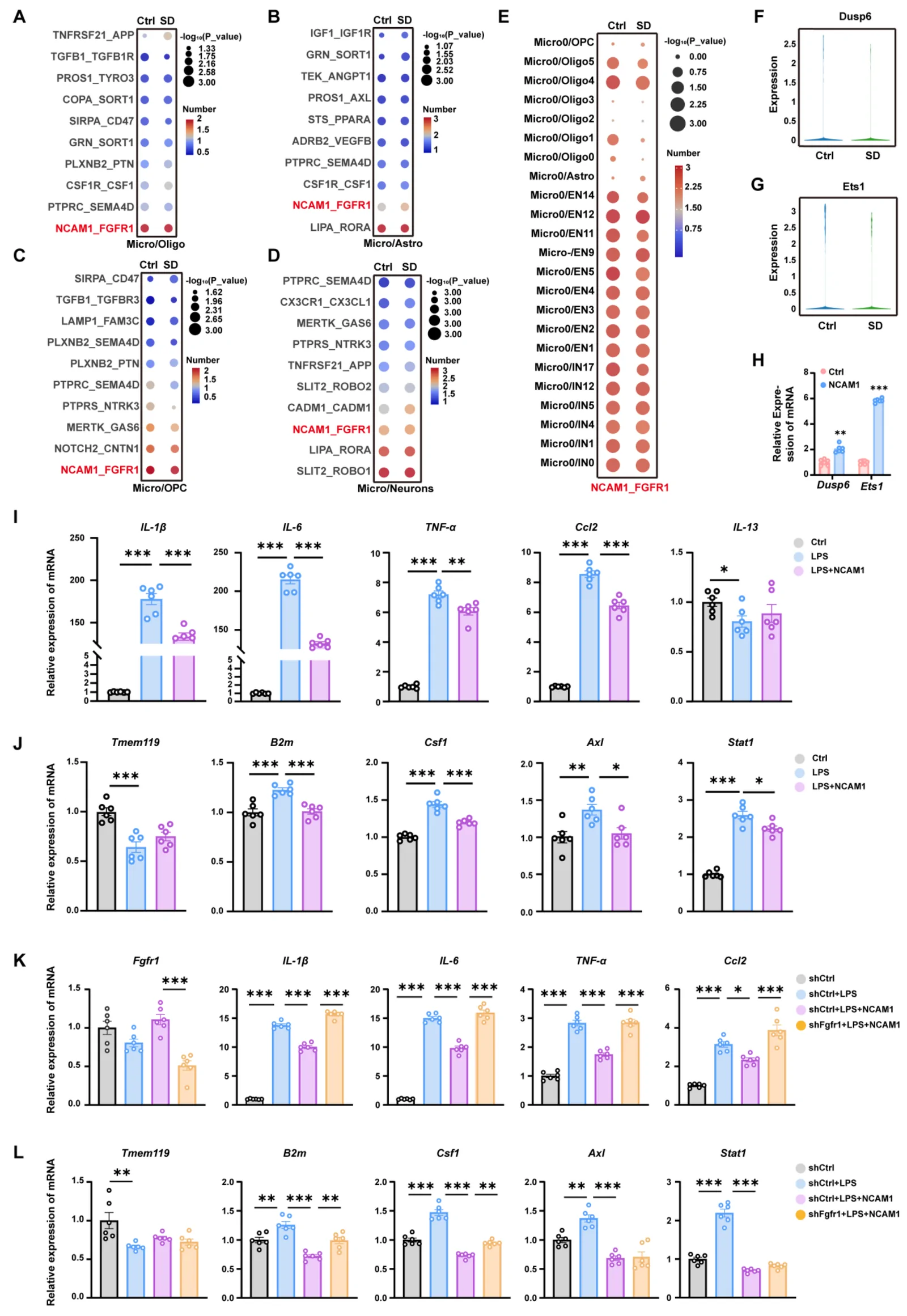
NCAM1/FGFR1 signaling negatively regulates microglial inflammation (**A**–**D**). Cellular interaction strength between microglia and Oligo (**A**), Astro (**B**), OPC (**C**), and Neurons (**D**) in Ctrl and SD groups. (**E**) NCAM1/FGFR1 interaction strength between Micro0 and other cell types. (**F**,**G**) Downregulation of downstream signaling molecules *Dusp6* (**F**) and *Ets1* (**G**) following SD by snRNA-seq. (**H**) Recombinant NCAM1 activated *Dusp6* and *Ets1* expression in BV2 cells. (**I**,**J**) NCAM1 attenuated LPS-induced pro-inflammatory cytokines (**I**) and restored homeostatic microglial markers (**J**). (**K**,**L**) Fgfr1 knockdown abolished the protective effects of NCAM1 on inflammatory cytokines (**K**) and microglial phenotype (**L**). Data are presented as mean ± SEM. Significance is indicated as * *p* < 0.05, ** *p* < 0.01, and *** *p* < 0.001.

To further investigate how SD modulates hypothalamic neuroinflammatory responses, we analyzed the intercellular communication network between microglia and other cell clusters. Among the top-ranked ligand–receptor pairs in the intercellular communication analysis, NCAM1/FGFR1 emerged as a prominent pathway facilitating communication between microglia and several cell types, including Oligo, Astro, OPC, and Neurons (Figure 5A–D). Although the interaction strength did not show a significant between-group difference, the prominent ranking of NCAM1/FGFR1 highlighted this pathway as a candidate for further mechanistic investigation. Given the more pronounced response of IN to SD, we next examined whether NCAM1/FGFR1 signaling varied among inhibitory neuronal subclusters. Nevertheless, no significant intergroup differences were seen in the NCAM1/FGFR1-mediated interaction strength between Micro0 and other cell subclusters (Figure 5E). Despite its high ranking in the interaction analysis, SD did not yield significant changes in the expression of the ligand or receptor. In Oligo, Astro, OPC, and Neurons, NCAM1 expression was comparable between SD and control groups; similarly, FGFR1 expression in microglia showed no significant difference (Figure S6A–E). Consistently, cell–cell interaction analysis showed no statistically significant difference in NCAM1/FGFR1 interaction strength between the SD and the Ctrl groups (Figure S6F). The species conservation analysis revealed similar conserved domains of NCAM1 and FGFR1 across mouse, rat, and human (Figure S6G,H). However, snRNA-seq analysis revealed that downstream targets of the FGFR1 signaling pathway, including *Dusp6* and *Ets1* [47], were downregulated in the SD group compared with the Ctrl group (Figure 5F,G). Furthermore, functional validation in BV2 cells showed that recombinant NCAM1 promoted the expression of *Dusp6* and *Ets1* (Figure 5H). These findings from the rat snRNA-seq discovery dataset suggested that although SD does not considerably alter the NCAM1/FGFR1 interaction at the ligand–receptor level, it may attenuate signaling output downstream of this pathway.

To investigate the functional role of the NCAM1/FGFR1 interaction in microglial inflammation under conditions relevant to SD-induced neuroinflammation, in vitro experiments were performed using BV2 microglia. LPS treatment markedly induced pro-inflammatory responses in BV2 cells, evidenced by a pronounced elevation of *IL-1β*, *TNF-α*, *IL-6*, and *Ccl2* mRNA levels, which was notably attenuated by subsequent treatment with recombinant NCAM1 (Figure 5I). Compared to the Ctrl group, LPS treatment markedly decreased the mRNA expression of the homeostatic microglial marker *Tmem119*, while concurrently increasing the DAM-associated genes *Axl*, *Csf1*, and *B2m*, along with the pro-inflammatory marker *Stat1*. Notably, co-treatment with NCAM1 mitigated these LPS-induced changes, restoring the expression levels of most genes to baseline (Figure 5J). These results indicated that recombinant NCAM1 inhibits LPS-induced DAM-like phenotypes (pro-inflammatory) in BV2 microglia.

To determine whether these effects are dependent on FGFR1, *Fgfr1* was knocked down in BV2 cells. The efficacy of *Fgfr1* knockdown was confirmed by reduced FGFR1 protein levels in the shFgfr1 group relative to the shCtrl group (Figure S6I,J). Notably, *Fgfr1* knockdown abolished the protective effects of NCAM1 against LPS-induced microglial activation. Co-treatment with LPS+NCAM1 following *Fgfr1* knockdown failed to suppress the LPS-induced elevation of pro-inflammatory cytokines *IL-1β*, *TNF-α*, *IL-6*, and *Ccl2,* compared wtih the LPS+NCAM1. Moreover, *Fgfr1* loss prevented NCAM1 from restoring the homeostatic marker Tmem119, whereas the expression of DAM-associated genes (*B2m*, *Csf1*, and *Axl*) and the pro-inflammatory marker *Stat1* remained significantly elevated (Figure 5K,L). These findings indicated that NCAM1 alleviates pro-inflammatory responses and restores microglial homeostasis in an *Fgfr1*-dependent manner.

### 3.6. Inhibition of the NCAM1/FGFR1 Axis Exacerbates Microglial Inflammatory Responses and Neuronal Injury

Given the role of the PVN in sleep–wake regulation, *Fgfr1* was knocked down in the mouse PVN using AAV-mediated shRNA. After a 3-week postoperative recovery period, mice were subjected to the SD2W protocol (Figure S7A).

Histological analysis showed more severe neuronal abnormalities in the shFgfr1+SD group than in the shCtrl+SD group. H&E staining revealed more morphologically abnormal cells in the hypothalamus, characterized by nuclear fragmentation, pyknosis, and eosinophilic cytoplasmic changes (Figure S7B,C). Consistently, Nissl staining showed a reduction in Nissl-body cells (Figure S7D,E). Following *Fgfr1* knockdown, NeuN immunofluorescence further showed fewer NeuN^+^ cells in the hypothalamus (Figure S7F,G). When combined, these findings indicate that PVN-targeted *Fgfr1* knockdown aggravated SD-associated neuronal injury.

*Fgfr1* knockdown was also associated with clear changes in microglial morphology. Compared with the shCtrl+SD group, microglia in the shFgfr1+SD group showed increased cell density and soma size, together with reduced process complexity and shorter maximal branch length (Figure S7H–M), consistent with a more activated microglial phenotype.

qPCR analysis further showed increased expression of multiple inflammatory mediators in the hypothalamus of shFgfr1+SD mice, including the pro-inflammatory cytokines *IL-1β*, *IL-6*, *TNF-α*, and *IFN-γ*; the chemokines *Ccl2*, *Ccl3*, *Ccl4*, *Ccl5*, *Cxcl1*, *Cxcl9*, *Cxcl10*, and *Cxcl11*; and the matrix metalloproteinases *Mmp3*, *Mmp9*, and *Mmp12* (Figure S7N).

Thus, non-cell-type-specific *Fgfr1* knockdown in the PVN exacerbated SD-associated neuronal injury, microglial activation, and neuroinflammatory responses in the hypothalamus. These findings support a protective contribution of endogenous FGFR1 signaling during SD and complement the in vitro evidence for *Fgfr1*-dependent regulation by NCAM1. Because *Fgfr1* was not manipulated in a cell-type-specific manner and knockdown was performed before SD exposure, these experiments do not identify the cellular population responsible for the effect or establish therapeutic efficacy.

These data collectively demonstrated that NCAM1 exerts its protective effects on microglia through an *Fgfr1*-dependent mechanism. This finding identified the NCAM1/FGFR1 pathway as a potential therapeutic target for alleviating hypothalamic neuronal injury brought on by neuroinflammation.

## 4. Discussion

This study suggests that altered downstream signaling within the NCAM1/FGFR1 pathway may be the mechanism of SD-induced hypothalamus injury. Our findings support the hypothesis that SD impaires protective signaling downstream of the NCAM1/FGFR1 pathway, coinciding with microglial activation and pro-inflammatory state, which could represent one mechanism underlying reduced neuronal markers. This work extends the observation of SD-associated inflammation by identifying a particular potential mechanism facilitating communication between microglia and other cell types. To the best of our knowledge, this study is among the first to implicate NCAM1/FGFR1 as a candidate neuroimmune communication pathway in SD.

While our study focused on the hypothalamus due to its central role in sleep–wake regulation, SD is known to affect multiple brain regions. Previous studies have reported microglial activation and neuroinflammation in the hippocampus, prefrontal cortex, amygdala, and thalamus following sleep deprivation [10,14,15,25,26,27,48], with some also documenting neuronal injury and altered intracellular signaling [14,27]. Notably, the downstream NCAM1/FGFR1 signaling molecules *Dusp6* and *Ets1* have been implicated in inflammatory responses [47,49], though whether the NCAM1/FGFR1 axis shows region-selective regulation remains unknown. Therefore, our findings should be interpreted as a characterization of the hypothalamic response to SD rather than evidence of hypothalamic specificity. The effects on other brain regions remain to be investigated in future studies.

A critical insight from our study is the identification of IN subpopulations as prominent neuronal populations that experience marked transcriptional alterations following SD. The relative reduction in IN0 and IN1 clusters suggested a selective vulnerability of these subpopulations at the transcriptomic level. Nonetheless, as this observation is derived from snRNA-seq proportional data, it does not constitute direct evidence of absolute cell loss. The hypothalamus is a key regulator of sleep–wake cycles, and the balance between excitatory and inhibitory signaling is crucial for proper circadian rhythms and sleep architecture [21]. Our analysis uncovered an increased proportion of IN and a decreased proportion of EN in the SD2W group, indicating the excitatory/inhibitory imbalance in the hypothalamic network. The transcriptional profile of IN0 indicated activation of neurodegenerative programs, including neuron death and axonogenesis.

We propose that the NCAM1/FGFR1 axis acts as a homeostatic brake on microglial inflammation. NCAM1, a neural adhesion molecule [50,51], mediates neuron–microglia crosstalk and supports neurite outgrowth and survival [52]. Previous studies have established a link between NCAM1/FGFR1 signaling and the inflammatory NF-κB [53] and ERK/MAPK pathways [54]. In vitro, recombinant NCAM1 suppressed LPS-induced BV2 microglial activation via *Fgfr1*. We interpret these findings as evidence that NCAM1/FGFR1 signaling has the capacity to modulate inflammatory activation in microglia. However, this in vitro model does not fully recapitulate the neuroinflammatory milieu induced by SD in vivo. In vivo, PVN *Fgfr1* knockdown exacerbated SD-induced microglial activation, neuroinflammation, and neuronal injury, while driving microglial transition to an amoeboid pro-inflammatory phenotype [55,56]. These complementary lines of evidence—across in vitro and in vivo systems, and across rat and mouse models—support a protective role for NCAM1/FGFR1 signaling in the hypothalamic response to SD. We found that NCAM1 activated the downstream effectors *Dusp6* and *Ets1*, suggesting that SD is associated with altered signaling output downstream of the NCAM1/FGFR1 pathway rather than a change in the expression of the complex itself or its ligand–receptor interaction strength.

The functional consequences of this disordered signaling are evident in the phenotypic shift in microglia, consistent with a prior report that glial cells are the most responsive to sleep deprivation [57]. This phenotypic transition is increasingly understood through DAM signatures [29,31]. Canonical DAM states are defined by downregulation of homeostatic genes (*P2ry12*, *Tmem119*, *Cx3cr1*) and upregulation of phagocytic/lipid metabolism genes (*Trem2*, *Apoe*, *Tyrobp*) [30,31,32]. Consistent with this, pseudotime analysis revealed an inferred trajectory toward a DAM1-like state, with upregulation of *Trem2*, *Itgam*, and *B2m* [55,56]. Pseudotime is a computational inference, not a demonstrated in vivo transition, and requires validation. In neurodegenerative diseases, dysregulated microglia release proinflammatory cytokines, reactive oxygen species, and reactive nitrogen species, which activate apoptotic signaling pathways and ultimately lead to neuronal death [58,59]. Microglia are the core executors of this process, with inflammatory factors such as *TNF-α*, *IL-1β*, and *IL-6* serving as key molecular mediators [14]. In alignment with this framework, our results demonstrated that SD-induced neuroinflammation is associated with reduced neuronal markers, supporting the notion that cytokine- and chemokine-mediated signals correlate with neuronal injury. The presence of this DAM1-like transcriptional pattern thus represents a pivotal feature linking SD to hypothalamic pathology, although confirmation of a genuine temporal transition awaits future longitudinal studies.

In addition to microglia, other glial cell types may also contribute to SD-induced neuropathology. These studies indicated that oligodendrocytes and their precursor cells actively participated in neuroinflammation and neuron death [60,61]. Furthermore, neuronal survival—particularly of energy-demanding axons—depends on metabolic support from oligodendrocytes [62]. Considering these findings, the possibility that oligodendrocytes contribute to SD-induced neuroinflammation and reduced neuronal markers cannot be excluded.

It is important to acknowledge several limitations of this study. First, the snRNA-seq experiment lacked independent biological replicates, as tissues from three rats were pooled into a single library per condition. Our transcriptomic findings should therefore be interpreted as exploratory and hypothesis-generating rather than confirmatory, and they require validation with independent biological replicates. Second, sleep architecture was not directly monitored using electroencephalography (EEG)/electromyography (EMG). The orbital rotation paradigm may induce stress, motor activity, and HPA axis activation, all of which can independently modulate neuroinflammation [63,64]. The PVN is particularly sensitive to both stress and sleep disruption [65]. The observed hypothalamic changes may reflect a convergence of sleep fragmentation, stress responses, and motor activity rather than sleep loss alone. Future studies incorporating EEG/EMG, stress hormone measurements, and appropriate control conditions are needed. Third, the exact molecular mechanism by which SD impairs NCAM1/FGFR1 signal transduction—whether through post-translational modifications, altered membrane microdomain localization, or co-receptor involvement—remains to be elucidated. Fourth, only male rodents were used; sex differences in sleep architecture, microglial biology, and inflammatory responses [66,67,68,69] mean our findings may not generalize to females. Future studies are therefore needed to determine whether these effects extend to females. Fifth, we used rats for snRNA-seq discovery and mice for functional validation; species-specific differences [70] mean these data should be viewed as complementary rather than directly integrated. Therefore, the rat and mouse data should be viewed as complementary rather than directly integrated. The conclusions drawn from cross-species integration are hypothesis-generating rather than confirmatory. Future studies using the same species across all components would strengthen causal interpretation. Sixth, PVN Fgfr1 knockdown was non-cell-type-specific and performed prior to SD, precluding identification of the responsible cell population and therapeutic efficacy assessment. Future cell-type-specific and temporally controlled studies are needed. Despite these limitations, our study establishes a clear link between NCAM1/FGFR1 dysfunction and SD-induced neuroinflammation, suggesting this axis as a potential therapeutic target.

## 5. Conclusions

These findings in male animals suggest a potential neuroimmune communication mechanism, as shown in the graphical abstract, and warrant further investigation into the NCAM1/FGFR1 pathway as a potential mechanistic target for sleep-related neurological disorders.

## Data Availability

The single-nucleus RNA-sequencing datasets generated and analyzed in this study will be publicly available in the Gene Expression Omnibus (GEO) repository under the accession number GSE342037 (https://www.ncbi.nlm.nih.gov/geo/query/acc.cgi?acc=GSE342037; accessed on 20 September 2026). Further inquiries can be directed to the corresponding authors.

## Supplementary Materials

The following supporting information can be downloaded at: https://www.mdpi.com/article/10.3390/cells15181716/s1, Figure S1: SD induces neuroinflammation in hypothalamus of mice. (A–F) mRNA expression levels of inflammation-related molecules Ccl2 (A), IL-4 (B), IL-10 (C), IL-13 (D), IL-17A (E), COX2 (F) of the Ctrl, SD1W and SD2W groups in hypothalamus of mice (n = 7/group). Data are presented as mean ± SEM. Significance is indicated as * *p* < 0.05, ** *p* < 0.01, and *** *p* < 0.001. Figure S2: snRNA-seq uncovers neuronal dysfunction in hypothalamus of rats. (A) Umap of IN and EN identified from the snRNA-seq data of hypothalamic tissues. Each cluster is shown with different color. (B) Heatmap showing the cell type-specific genes are differentially expressed in IN and EN cluster. (C) DEGs of EN cluster. (D) GO enrichment analysis of upregulated DEGs in EN cluster for BP. (E) DEGs of IN cluster. (F) GO enrichment analysis of upregulated DEGs in IN cluster for BP. (G) KEGG enrichment analysis of upregulated DEGs in EN cluster. (H) KEGG enrichment analysis of upregulated DEGs in IN cluster. Figure S3: snRNA-seq uncovers IN1 cluster dysfunction in hypothalamus of rats. (A) Heatmap showing the cell type-specific genes are differentially expressed in IN subclusters. (B) Heatmap showing differentiated genes in different signaling pathway. (C) DEGs of IN1 cluster. (D) GO enrichment analysis of downregulated DEGs in IN1 cluster for cellular component. (E) GSEA showing the enriched KEGG pathway in IN1 cluster. Figure S4: SD induces neuroinflammation response in Micro1. (A) DEGs of Micro1 cluster. (B) Heatmap showing the cell type-specific genes are differentially expressed in microglia clusters. (C) GO enrichment analysis of upregulated DEGs in Micro1 cluster for BP. (D) KEGG enrichment of upregulated DEGs in Micro1 cluster. Figure S5: SD induces neuroinflammation response in Micro. (A) GO enrichment analysis of upregulated DEGs in Micro cluster for BP. (B) KEGG enrichment of upregulated DEGs in Micro cluster. (C) GO enrichment analysis of downregulated DEGs in Micro cluster for BP. (D) DAM2 gene set score between the Ctrl and SD groups. Figure S6: Cell interaction analysis related NCAM1/FGFR1 interaction. (A–D) Ncam1 expression in Oligo, Astro, OPC and Neuron clusters between the Ctrl and SD groups. (E) Fgfr1 expression in microglia between the Ctrl and SD groups. (F) NCAM1/FGFR1 interaction strength of Micro-Neuron, Micro-Astro, Micro-OPC, and Micro-Oligo between the Ctrl and SD groups. (G,H) The species conservation analysis of NCAM1 (G) and FGFR1 (H). Figure S7: NCAM1/FGFR1 axis exacerbates microglial inflammatory responses and neuronal injury via shFgfr1 in mice. (A) Schematic representation of stereotaxic injection of AAV9-expressing Fgfr1 targeted to the PVN in male mice. (B) H&E staining of hypothalamic sections between shCtrl+SD and shFgfr1+SD groups. Scale bars: left panel, 50 μm and rght panel, 20 μm. 3V, third ventricle. (C) Semi-quantitative analysis of morphologically abnormal cells (n = 3/group). (D) Nissl staining of hypothalamic sections (scale bars same as in B). (E) Quantification of nissl bodies (n = 3/group). (F) Representative IF staining for NeuN+ cells. Scale bars: left panel, 50 μm, and right panel, 20 μm. (G) Quantitative analysis of NeuN+ cells (n = 3/group). (H) Representative IF staining for IBA1+ cells (scale bars same as in F). (I) The binary and skeleton of microglia. (J–M) Diagram of quantification of microglia (n = 3/group). (N) mRNA levels of inflammation-related molecules in the mouse hypothalamus between the shCtrl+SD and shFgfr1+SD groups (n = 7/group).Data are presented as mean ± SEM. Significance is indicated as * *p* < 0.05, ** *p* < 0.01, and *** *p* < 0.001.

## Abbreviations

SD	Sleep disturbance
snRNA-seq	Single-nucleus RNA sequencing
NCAM1	Neural cell adhesion molecule 1
FGFR1	Fibroblast growth factor receptor 1
DEGs	Differentially expressed genes
DAM	Disease-associated microglia
GSEA	Gene set enrichment analysis
IF	Immunofluorescence
CNS	Central nervous system
EN	Excitatory neurons
IN	Inhibitory neurons
Micro	Microglia
Oligo	Oligodendrocyte
OPC	Oligodendrocyte precursor cells
FIbro	Fibroblasts
Astro	Astrocytes
Endo	Endothelial cells
LPS	Lipopolysaccharide
PVN	Paraventricular nucleus

## Appendix A

**Table A1:** Primer sequence (mouse).

Primers	Forward Primer	Reverse Primer
*IL-6*	TAGTCCTTCCTACCCCAATTTCC	TTGGTCCTTAGCCACTCCTTC
*IL-1β*	GCAACTGTTCCTGAACTCAACT	ATCTTTTGGGGTCCGTCAACT
*TNFα*	GACGTGGAACTGGCAGAAGAG	TTGGTGGTTTGTGAGTGTGAG
*Ccl2*	CCTTTGAATGTGAAGTTGACCCGTAAATC	GAAGTGCTTGAGGTGGTTGTGGAA
*IL-17A*	ATGCTGTTGCTGCTGCTGAG	GTGGAACGGTTGAGGTAGTCTG
*COX2*	TTCAACACACTCTATCACTGGC	AGAAGCGTTTGCGGTACTCAT
*IL-4*	GGTCTCAACCCCCAGCTAGT	GCCGATGATCTCTCTCAAGTGAT
*IL-10*	GCTCTTACTGACTGGCATGAG	CGCAGCTCTAGGAGCATGTG
*IL-13*	TCTTGCTTGCCTTGGTGGTCTC	AGTCTGGTCTTGTGTGATGTTGCT
*Ets1*	AGTTTCAGCCATCACAACACA	GAAATCCTACCTGACGAGCAC
*Dusp6*	ATAGATACGCTCAGACCCGTG	ATCAGCAGAAGCCGTTCGTT
*Tmem119*	CCTACTCTGTGTCACTCCCG	CACGTACTGCCGGAAGAAATC
*B2m*	TTCTGGTGCTTGTCTCACTGA	CAGTATGTTCGGCTTCCCATTC
*Csf1*	ATGAGCAGGAGTATTGCCAAGG	TCCATTCCCAATCATGTGGCTA
*Axl*	ATGGCCGACATTGCCAGTG	CGGTAGTAATCCCCGTTGTAGA
*Stat1*	TCACAGTGGTTCGAGCTTCAG	GCAAACGAGACATCATAGGCA
*Fgfr1*	ATACCACCTACTTCTCCGTCAATGTCT	GTCATCGTCGTCGTCATCATCTTCC
*Gapdh*	AGGTCGGTGTGAACGGATTTG	TGTAGACCATGTAGTTGAGGTCA
*Cd86*	TTGATAGTGTGAATGCCAAGTACCT	ATTGTGAAGTCGTAGAGTCCAGTTG
*IFN-γ*	AACTCAAGTGGCATAGATGTGGAAGAA	CAGGTGTGATTCAATGACGCTTATGTT
*Ccr2*	TTCGCTGTAGGAATGAGAAGAAGAGG	TGGAAGGTGGTCAAGAAGAGAACAAT
*Ccl3*	TTCTCTGTACCATGACACTCTGC	CGTGGAATCTTCCGGCTGTAG
*Ccl4*	TTCCTGCTGTTTCTCTTACACCT	CTGTCTGCCTCTTTTGGTCAG
*Ccl5*	GCTGCTTTGCCTACCTCTCC	TCGAGTGACAAACACGACTGC
*Cxcl1*	ATGGCTGGGATTCACCTCAAGAAC	GTGTGGCTATGACTTCGGTTTGG
*Cxcl9*	CATTGCTACACTGAAGAACGGAGAT	CCTTGAACGACGACGACTTTGG
*Cxcl10*	CCAAGTGCTGCCGTCATTTTC	GGCTCGCAGGGATGATTTCAA
*Cxcl11*	GGCTTCCTTATGTTCAAACAGGG	GCCGTTACTCGGGTAAATTACA
*Mmp3*	ACATGGAGACTTTGTCCCTTTTG	TTGGCTGAGTGGTAGAGTCCC
*Mmp9*	CTGGACAGCCAGACACTAAAG	CTCGCGGCAAGTCTTCAGAG
*Mmp12*	AACAACTTAGTACCAGAGCCACACT	TGCCTCACATCATACCTCCAGTAGT

**Table A2:** Genes and their function (rats).

Gene	Function
*IL-17*	Pro-inflammatory cytokine that regulates immune responses and inflammation
*Mmp2*	Degrades extracellular matrix components; involved in tissue remodeling and inflammation
*Cst3*	Cysteine protease inhibitor; regulates proteolysis and is involved in cellular stress responses
*Hexb*	Lysosomal enzyme involved in glycolipid metabolism
*Cx3cr1*	Chemokine receptor mediating microglia-neuron communication; regulates cell migration and signaling
*Trem2*	Myeloid cell receptor that activates microglial phagocytosis and immune responses
*B2m*	Component of MHC class I complex; essential for antigen presentation
*Itgam*	Integrin subunit (CD11b) that mediates leukocyte adhesion and migration
*Fkbp5*	Peptidyl-prolyl isomerase involved in protein folding and glucocorticoid signaling
*Ccnd3*	Cyclin that promotes G1/S cell cycle progression and cell proliferation
*Pten*	Phosphatase that negatively regulates PI3K/AKT signaling; controls cell growth and survival
*C1qa*	Complement C1q components that activate the classical complement pathway and mediate synapse pruning
*C1qb*	Complement C1q components that activate the classical complement pathway and mediate synapse pruning
*C1qc*	Complement C1q components that activate the classical complement pathway and mediate synapse pruning
*Psap*	Precursor of saposins; facilitates lysosomal sphingolipid breakdown
*Ctss*	Lysosomal cysteine protease involved in antigen processing and presentation
*Sparc*	Secreted matrix protein that modulates cell–matrix interactions and tissue remodeling
*Ptgds*	Synthesizes prostaglandin D2; regulates inflammation and immune responses
*Csf1r*	Receptor for M-CSF; critical for microglial survival, proliferation, and differentiation
*Hck*	Src-family tyrosine kinase that transduces signals in myeloid cells
*Cd84*	Immune cell surface receptor involved in cell–cell adhesion and signaling
*Lyn*	Src-family tyrosine kinase that regulates signaling in B cells and myeloid cells
*Irf3*	Transcription factor that activates type I interferon responses to viral infection
*Mertk*	Receptor tyrosine kinase that mediates phagocytic clearance of apoptotic cells
*P2ry12*	Purinergic receptor highly expressed in resting microglia; a mature microglial marker
*Tmem119*	Transmembrane protein specifically expressed in homeostatic microglia; a highly specific microglial marker
*Tyrobp*	Adaptor protein that transmits activating signals from TREM2 and other myeloid receptors
*Apoe*	Lipid transporter; upregulated in reactive glia following CNS injury
*Lpl*	Lipoprotein lipase; hydrolyzes triglycerides in lipid metabolism
*Axl*	Receptor tyrosine kinase involved in cell survival, proliferation, and efferocytosis
*Cd9*	Tetraspanin that regulates cell adhesion, migration, and membrane organization
*Spp1*	Secreted phosphoprotein that modulates inflammation and extracellular matrix remodeling
*Cst7*	Cysteine protease inhibitor expressed in myeloid cells
*Itgax*	Integrin subunit (CD11c); a marker for dendritic cells and activated microglia
*Dusp6*	Phosphatase that negatively regulates MAPK signaling pathways
*Ets1*	Transcription factor that controls gene expression during immune cell differentiation
