# Supplementary material for: Targeting NCAM1/FGFR1 Signaling to Alleviate Sleep Disturbance-Induced Neuroinflammation in the Hypothalamus
Source: Cells. 2026 Sep 21;15(18):1716. doi: 10.3390/cells15181716 (PMC13605233; doi:10.3390/cells15181716)
Supplement: Supplementary file 1 [file cells-15-01716-s001.zip › cells-4513871-supplementary.pdf]

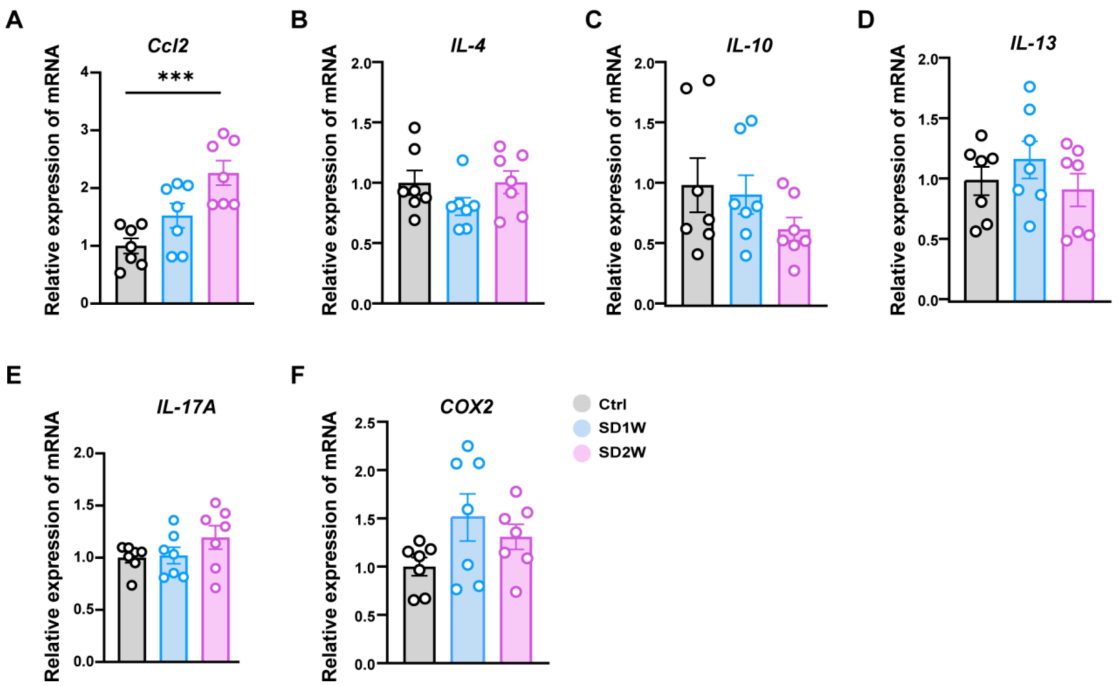

Figure S1. SD induces neuroinflammation in hypothalamus of mice. (A-F) mRNA expression levels of inflammation-related molecules *Ccl2* (A), *IL-4* (B), *IL-10* (C), *IL-13* (D), IL-17A (E), COX2 (F) of the Ctrl, SD1W and SD2W groups in hypothalamus of mice (n = 7/group). Data are presented as mean ± SEM. Significance is indicated as * $p < 0.05$, ** $p < 0.01$, and *** $p < 0.001$.

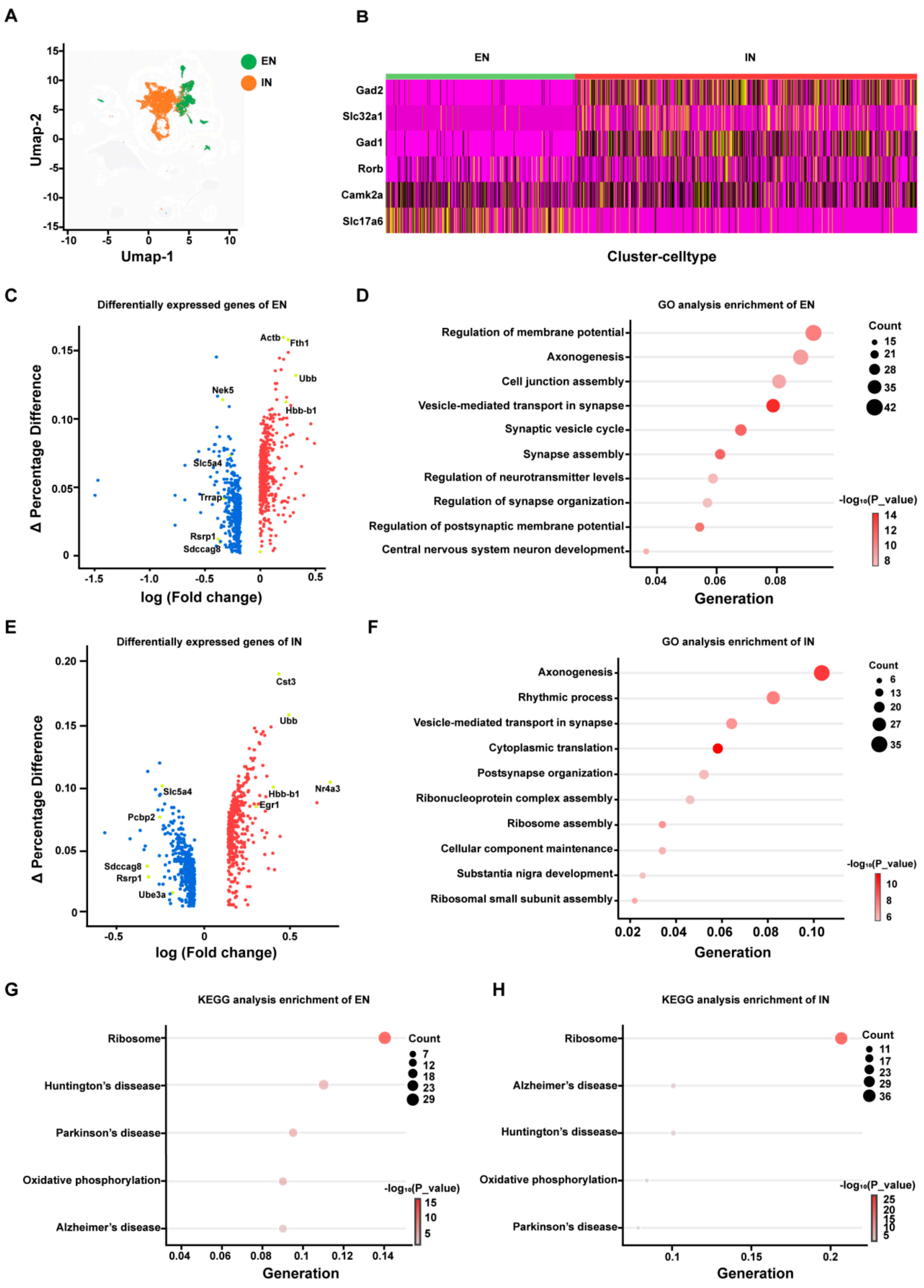

Figure S2. snRNA-seq uncovers neuronal dysfunction in hypothalamus of rats. (A) Umap of IN and EN identified from the snRNA-seq data of hypothalamic tissues. Each cluster is shown with different color. (B) Heatmap showing the cell type-specific genes are differentially expressed in IN and EN cluster. (C) DEGs of EN cluster. (D) GO enrichment analysis of upregulated DEGs in EN cluster for BP. (E) DEGs of IN cluster. (F) GO enrichment analysis of upregulated DEGs in IN cluster for BP. (G) KEGG enrichment analysis of upregulated DEGs in EN cluster. (H) KEGG enrichment analysis of upregulated DEGs in IN cluster.

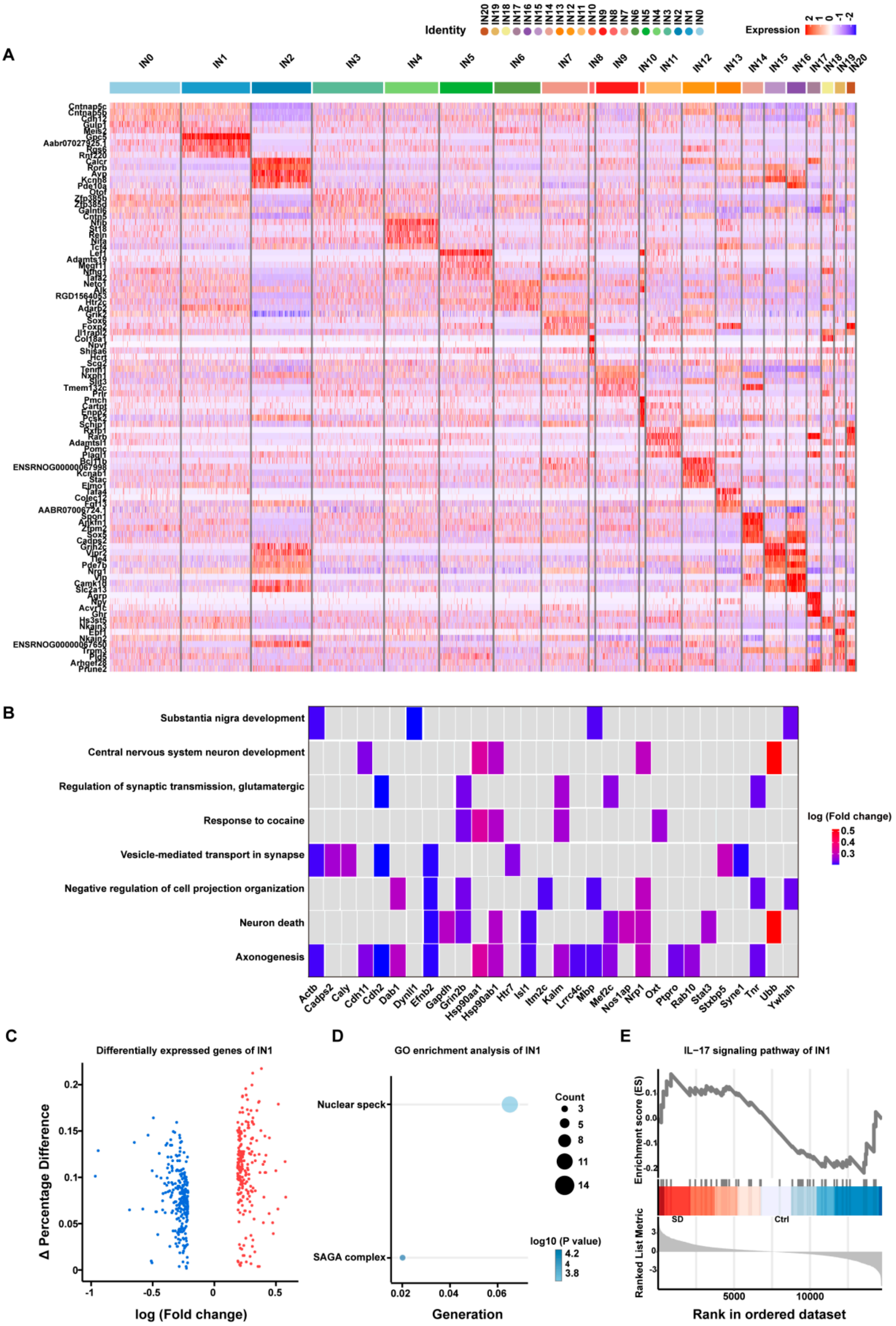

Figure S3. snRNA-seq uncovers IN1 cluster dysfunction in hypothalamus of rats. (A) Heatmap showing the cell type-specific genes are differentially expressed in IN subclusters. (B) Heatmap showing differentiated genes in different signaling pathway. (C) DEGs of IN1 cluster. (D) GO enrichment analysis of downregulated DEGs in IN1 cluster for cellular component. (E) GSEA showing the enriched KEGG pathway in IN1 cluster.

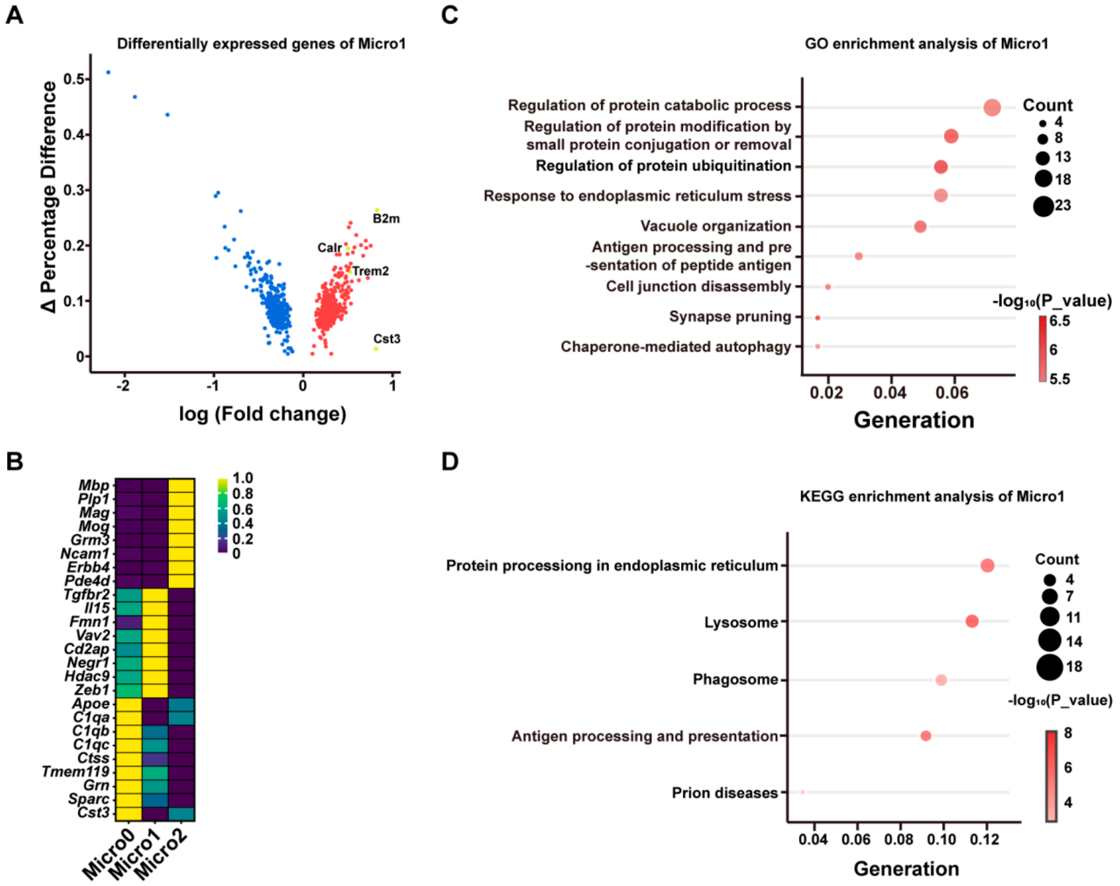

Figure S4. SD induces neuroinflammation response in Micro1. (A) DEGs of Micro1 cluster. (B) Heatmap showing the cell type-specific genes are differentially expressed in microglia clusters. (C) GO enrichment analysis of upregulated DEGs in Micro1 cluster for BP. (D) KEGG enrichment of upregulated DEGs in Micro1 cluster.

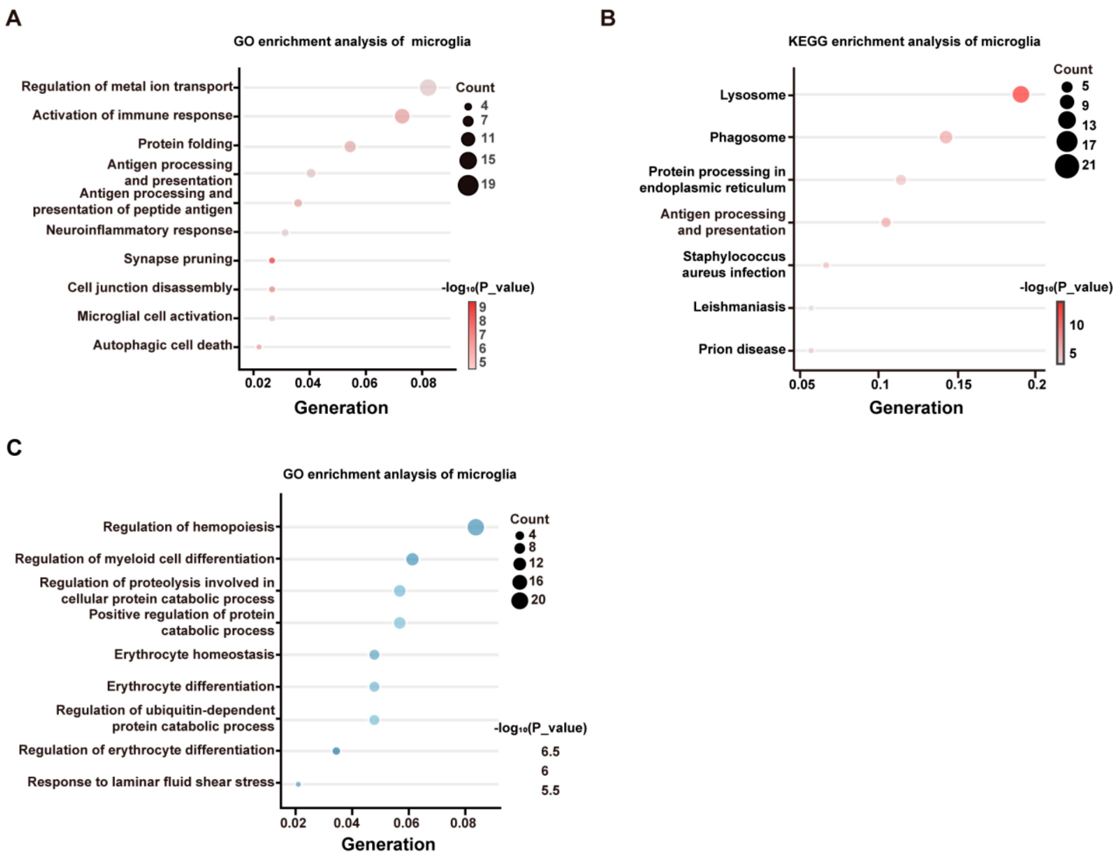

Figure S5. SD induces neuroinflammation response in Micro. (A) GO enrichment analysis of upregulated DEGs in Micro cluster for BP. (B) KEGG enrichment of upregulated DEGs in Micro cluster. (C) GO enrichment analysis of downregulated DEGs in Micro cluster for BP. (D) DAM2 gene set score between the Ctrl and SD groups.

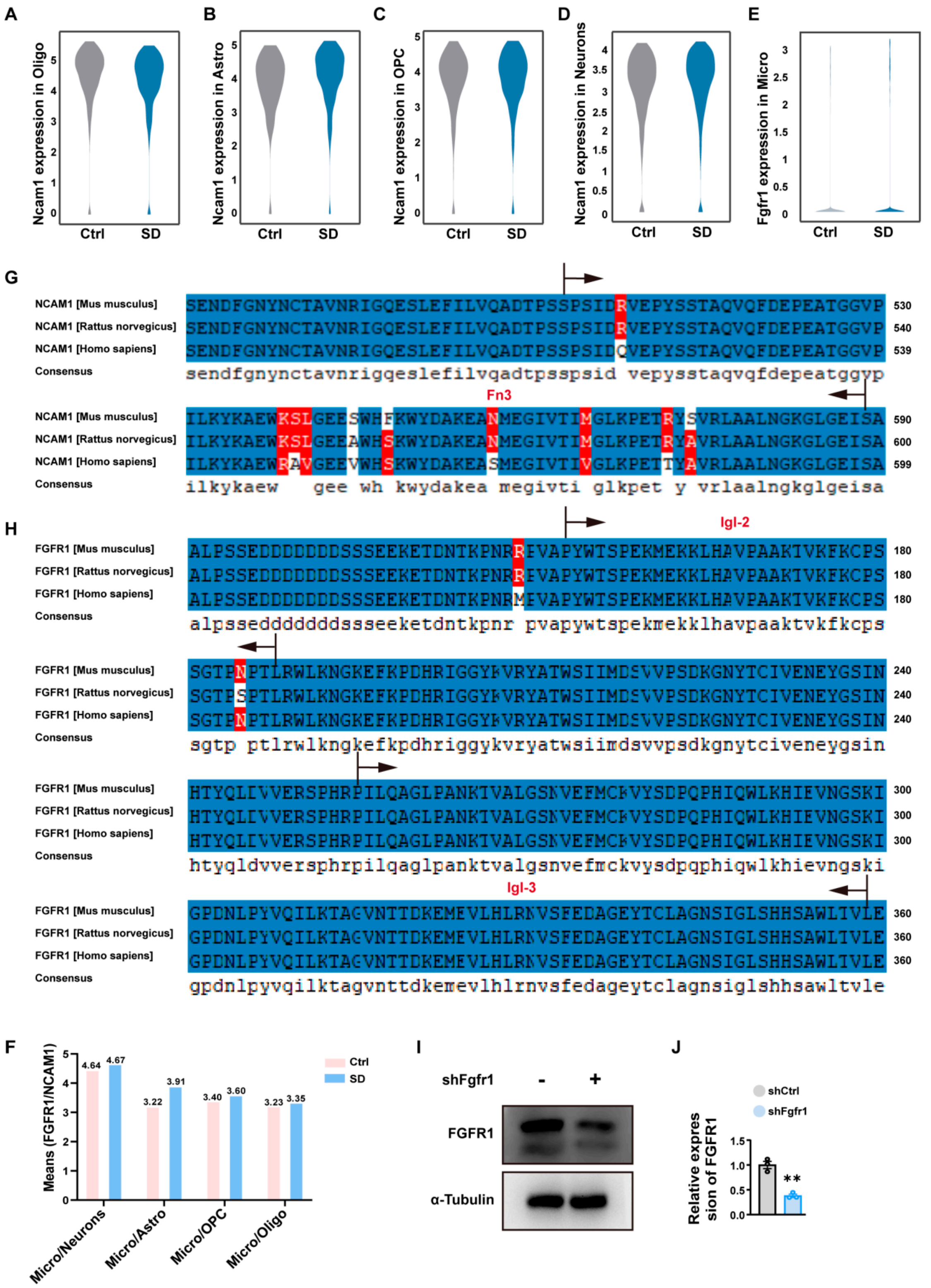

Figure S6. Cell interaction analysis related NCAM1/FGFR1 interaction. (A-D) Ncam1 expression in Oligo, Astro, OPC and Neuron clusters between the Ctrl and SD groups. (E) Fgfr1 expression in microglia between the Ctrl and SD groups. (F) NCAM1/FGFR1 interaction strength of Micro-Neuron, Micro-Astro, Micro-OPC, and Micro-Oligo between the Ctrl and SD groups. (G-H) The species conservation analysis of NCAM1 (G) and FGFR1 (H).

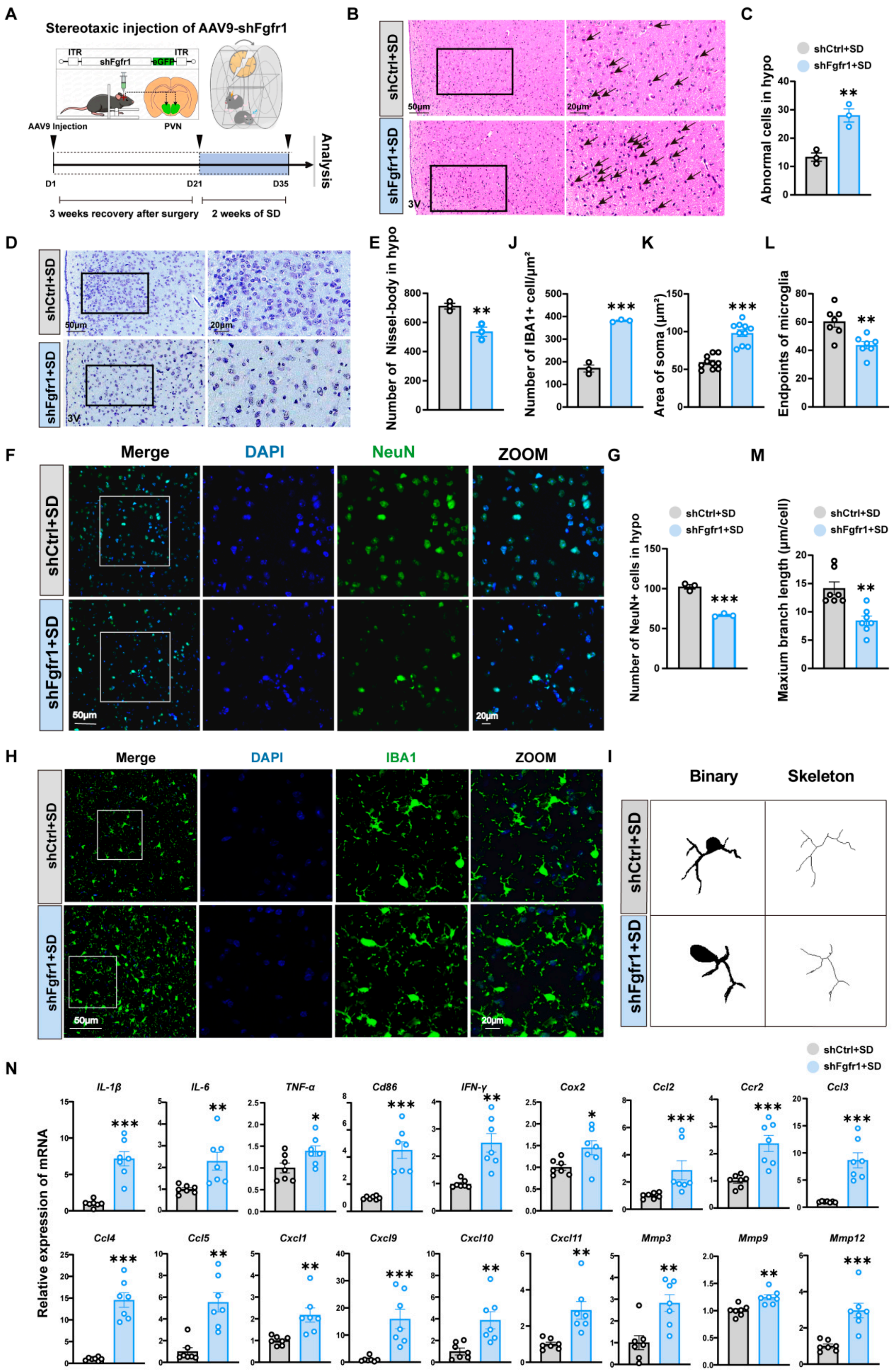

Figure S7. NCAM1/FGFR1 axis exacerbates microglial inflammatory responses and neuronal injury via shFgfr1 in mice. (A) Schematic representation of stereotaxic injection of AAV9-expressing *Fgfr1* targeted to the PVN in male mice. (B) H&E staining of hypothalamic sections between shCtrl+SD and shFgfr1+SD groups. Scale

bars: left panel, 50 μm and rght panel, 20 μm. 3V, third ventricle. (C) Semi-quantitative analysis of morphologically abnormal cells (n = 3/group). (D) Nissl staining of hypothalamic sections (scale bars same as in B). (E) Quantification of nissl bodies (n = 3/group). (F) Representative IF staining for NeuN+ cells. Scale bars: left panel, 50 μm, and right panel, 20 μm. (G) Quantitative analysis of NeuN+ cells (n = 3/group). (H) Representative IF staining for IBA1+ cells (scale bars same as in F). (I) The binary and skeleton of microglia. (J-M) Diagram of quantification of microglia (n = 3/group). (N) mRNA levels of inflammation-related molecules in the mouse hypothalamus between the shCtrl+SD and shFgfr1+SD groups (n = 7/group).Data are presented as mean ± SEM. Significance is indicated as $*p < 0.05$, $**p < 0.01$, and $***p < 0.001$.
